# A repeat expansion in *GOLGA8A* is a major risk factor for atypical frontotemporal lobar degeneration with ubiquitin-positive inclusions

**DOI:** 10.1038/s41588-026-02537-7

**Published:** 2026-03-12

**Authors:** Wouter De Coster, Marleen Van den Broeck, Matt Baker, Nikhil B. Ghayal, Sarah Wynants, Anthony Batzler, Cyril Pottier, Sara Alidadiani, Fahri Küçükali, Gregory D. Jenkins, Rafaela Policarpo, Marka van Blitterswijk, Mariely DeJesus-Hernandez, Alexandra I. Soto-Beasley, Júlia Faura, Elise Coopman, Saskia Hutten, Merel O. Mol, David Wallon, Anne Sieben, Elizabeth C. Finger, Melissa E. Murray, Shelley L. Forrest, Maria C. Tartaglia, Claire Troakes, Jeroen G. J. van Rooij, Aivi T. Nguyen, R. Ross Reichard, Natalie L. Woodman, Alissa L. Nana, Sandra Weintraub, Tamar Gefen, Bart De Vil, Istvan Bodi, Oscar L. Lopez, Susana Boluda, Serge Belliard, Florence Lebert, Florent Marguet, Qinwen Mao, Marsel M. Mesulam, Adam L. Boxer, Mathieu Vandenbulcke, EunRan Suh, Jolien Schaeverbeke, Jean-Charles Lambert, Sonja W. Scholz, Clifton L. Dalgard, Bryan J. Traynor, Raphael J. Gibbs, Gerard D. Schellenberg, Dorothee Dormann, Geert Joris, Tim De Pooter, Peter De Rijk, Svenn D’Hert, Jasper Van Dongen, Julie van der Zee, Mojca Strazisar, Marla Gearing, Thomas Kukar, Margaret Flanagan, Sebastiaan Engelborghs, Bernardino Ghetti, Kathy L. Newell, Andrew King, Sigrun Roeber, Howard J. Rosen, Salvatore Spina, Patrick Cras, Nilüfer Ertekin-Taner, Zbigniew K. Wszolek, Ryan J. Uitti, William P. Cheshire, Wolfgang Singer, Jochen Herms, Keith A. Josephs, Jennifer L. Whitwell, Ronald C. Petersen, Florence Pasquier, Gaël Nicolas, Rudolph Castellani, Jonathan Glass, Bruce L. Miller, Gabor G. Kovacs, Robert A. Rissman, Annie Hiniker, Vincent Deramecourt, Lee-Cyn Ang, Jin Lee-Way, Vivianna M. Van Deerlin, Brittany N. Dugger, Dietmar R. Thal, Lea T. Grinberg, Carlos Cruchaga, Thomas Arzberger, David G. Munoz, Julia Keith, Lorne Zinman, Ekaterina Rogaeva, Edward B. Lee, Stephen J. Haggarty, Olaf Ansorge, Masud Husain, Glenda M. Halliday, Safa Al-Sarraj, Owen A. Ross, Kristel Sleegers, Rik Vandenberghe, Bradley F. Boeve, Neill R. Graff-Radford, Julia Kofler, Charles L. White, Tammaryn Lashley, Manuela Neumann, Joanna M. Biernacka, William W. Seeley, Harro Seelaar, John C. van Swieten, Jonathan D. Rohrer, Dennis W. Dickson, Ian R. A. Mackenzie, Rosa Rademakers

**Affiliations:** 1 Department of Biomedical Sciences, University of Antwerp, Antwerp, Belgium; 2 VIB Center for Molecular Neurology, VIB, Antwerp, Belgium; 3 Department of Neuroscience, Mayo Clinic, Jacksonville, FL USA; 4 Department of Quantitative Health Sciences, Mayo Clinic, Rochester, MN USA; 5 Department of Neurology, Washington University School of Medicine, St. Louis, MO USA; 6 NeuroGenomics and Informatics Center, Washington University School of Medicine, St. Louis, MO USA; 7 Biocenter, Institute of Molecular Physiology, Johannes Gutenberg-Universität, Mainz, Germany; 8 Department of Clinical Genetics, Erasmus Medical Center, Rotterdam, the Netherlands; 9 Univ Rouen Normandie, Inserm U1245 and CHU Rouen, Department of Neurology and CNRMAJ, Rouen, France; 10 Laboratory of Neurology, Translational Neuroscieces, Faculty of Medicine and Health Sciences, University of Antwerp, Antwerp, Belgium; 11 Neuropathology lab, IBB-NeuroBiobank BB1901113, Born Bunge Institute, Antwerp, Belgium; 12 Department of Pathology, Antwerp University Hospital – UZA, Antwerp, Belgium; 13 Department of Clinical Neurological Sciences, Schulich School of Medicine and Dentistry, University of Western Ontario, London, Ontario Canada; 14 Department of Laboratory Medicine and Pathology, Mayo Clinic, Jacksonville, FL USA; 15 Laboratory Medicine Program & Krembil Brain Institute, University Health Network, Toronto, Ontario Canada; 16 Tanz Centre for Research in Neurodegenerative Disease, University of Toronto, Toronto, Ontario Canada; 17 University Health Network Memory Clinic, Krembil Brain Institute, Toronto, Ontario Canada; 18 London Neurodegenerative Diseases Brain Bank, Department of Basic and Clinical Neuroscience, Institute of Psychiatry, Psychology & Neuroscience, King’s College London, London, UK; 19 Department of Neurology, Erasmus Medical Center, Rotterdam, the Netherlands; 20 Department of Laboratory Medicine and Pathology, Mayo Clinic, Rochester, MN USA; 21 Queens Square Brain Bank, Institute of Neurology, UCL, London, UK; 22 Department of Neurology, UCSF Weill Institute for Neurosciences, University of California, San Francisco, San Francisco, CA USA; 23 Mesulam Institute for Cognitive Neurology and Alzheimer’s Disease, Northwestern University, Chicago, IL USA; 24 Department of Neurology, Antwerp University Hospital - UZA, Antwerp, Belgium; 25 Department of Clinical Neuropathology, King’s College Hospital NHS Foundation Trust, London, UK; 26 Department of Neurology, University of Pittsburgh, Pittsburgh, PA USA; 27 Sorbonne University, APHP, Department of Neuropathology, DMU-Neuroscience, University Hospital Pitié-Salpêtrière, Institut du Cerveau - Paris Brain Institute - ICM, Inserm U1127, Paris, France; 28 Normandie Univ, Unicaen, PSL Research University, EPHE, Inserm U1077, CHU de Caen, Neuropsychologie et Imagerie de la Mémoire Humaine, and service de Neurologie, CMRR Haute Bretagne, Chu Pontchaillou, Rennes, France; 29 Memory center, Department of Neurology, Lille University Hospital, Lille, France; 30 Univ Rouen Normandie, Inserm U1245 and CHU Rouen, Department of Pathology and Laboratoire d’Anatomie Pathologique, Rouen, France; 31 Department of Pathology, University of Utah, Salt Lake City, UT USA; 32 Department of Geriatric Psychiatry, University Hospitals Leuven (UZ Leuven), Leuven, Belgium; 33 Neuropsychiatry, Department of Neurosciences, Leuven Brain Institute, KU Leuven, Leuven, Belgium; 34 Department of Pathology and Laboratory Medicine, Perelman School of Medicine at the University of Pennsylvania, Philadelphia, PA USA; 35 Laboratory for Cognitive Neurology, Department of Neurosciences, Leuven Brain Institute, KU Leuven, Leuven, Belgium; 36 Laboratory of Neuropathology, Department of Imaging and Pathology, Leuven Brain Institute, KU Leuven, Leuven, Belgium; 37 Univ. Lille, Inserm, CHU Lille, U1167-RID-AGE facteurs de risque et déterminants moléculaires des maladies liés au vieillissement, Institut Pasteur de Lille, Lille, France; 38 Neurodegenerative Diseases Research Section, National Institute of Neurological Disorders and Stroke, Bethesda, MD USA; 39 Department of Neurology, Johns Hopkins University Medical Center, Baltimore, MD USA; 40 Department of Anatomy, Physiology and Genetics, Uniformed Services University of the Health Sciences, Bethesda, MD USA; 41 Neuromuscular Diseases Research Section, National Institute on Aging, Bethesda, MD USA; 42 Computational Biology Group, Laboratory of Neurogenetics, National Institute on Aging, Bethesda, MD USA; 43 Institute for Molecular Biology, Mainz, Germany; 44 Department of Pathology and Laboratory Medicine and Department of Neurology, Emory University, Atlanta, GA USA; 45 Department of Pharmacology and Chemical Biology, Emory University, Atlanta, GA USA; 46 University of Texas Health Science Center San Antonio, San Antonio, TX USA; 47 Department of Neurology, Universitair Ziekenhuis Brussel (UZ Brussel), Brussels, Belgium; 48 NEUR Research Group, Center for Neurosciences (C4N), Vrije Universiteit Brussel, Brussels, Belgium; 49 Department of Pathology and Laboratory Medicine, Indiana University School of Medicine, Indianapolis, IN USA; 50 Centre for Neuropathology and Prion Research, Ludwig-Maximilians-University of Munich, Munich, Germany; 51 Department of Pathology, UCSF Weill Institute for Neurosciences, University of California, San Francisco, San Francisco, CA USA; 52 Department of Neurology, Mayo Clinic, Jacksonville, FL USA; 53 Department of Neurology, Division of Autonomic Neurology, Mayo Clinic, Jacksonville, FL USA; 54 Department of Neurology, Mayo Clinic, Rochester, MN USA; 55 German Center for Neurodegenerative Diseases (DZNE), Munich, Germany; 56 Department of Radiology, Mayo Clinic, Rochester, MN USA; 57 Univ. Lille, Inserm, CHU Lille, LilNCog-Lille Neuroscience and Cognition, Lille, France; 58 Univ Rouen Normandie, Inserm U1245 and CHU Rouen, Department of Genetics and CNR-MAJ, Rouen, France; 59 Department of Pathology, Feinberg School of Medicine, Northwestern University, Chicago, IL USA; 60 Department of Laboratory Medicine and Pathobiology and Department of Medicine, University of Toronto, Toronto, Ontario Canada; 61 Edmond J. Safra Program in Parkinson’s Disease, Rossy PSP Centre and the Morton and Gloria Shulman Movement Disorders Clinic, Toronto Western Hospital, Toronto, Ontario Canada; 62 Department of Physiology and Neuroscience, Alzheimer’s Therapeutic Research Institute, Keck School of Medicine of the University of Southern California, San Diego, CA USA; 63 Department of Pathology, University of Southern California, Los Angeles, CA USA; 64 Department of Pathology and Laboratory Medicine, University of Western Ontario, London, Ontario Canada; 65 Department of Pathology, London Health Sciences Center, Western University, London, Ontario Canada; 66 Department of Pathology, University of California Davis Medical Center, Sacramento, CA USA; 67 Department of Pathology, University Hospital Leuven (UZ Leuven), Leuven, Belgium; 68 Department of Psychiatry, Knight Alzheimer Disease Research Center, Washington University School of Medicine, St. Louis, MO USA; 69 Department of Psychiatry and Psychotherapy, University Hospital, Ludwig-Maximilians-University of Munich, Munich, Germany; 70 St. Michael’s Hospital, Toronto, Ontario Canada; 71 Department of Laboratory Medicine and Pathobiology, University of Toronto, Toronto, Ontario Canada; 72 Sunnybrook Health Sciences Centre, Toronto, Ontario Canada; 73 Department of Neurology, Massachusetts General Hospital and Harvard Medical School, Boston, MA USA; 74 Nuffield Department of Clinical Neurosciences, University of Oxford, Oxford, UK; 75 University of Sydney, Faculty of Medicine and Health School of Medical Sciences and Brain and Mind Centre, Sydney, New South Wales Australia; 76 Department of Neurology, University Hospitals Leuven (UZ Leuven), Leuven, Belgium; 77 Department of Pathology, University of Pittsburgh, Pittsburgh, PA USA; 78 Division of Neuropathology, University of Texas Southwestern Medical Center, Dallas, TX USA; 79 Department of Neurodegenerative Disease, UCL Queen Square Institute of Neurology, London, UK; 80 Department of Neuropathology, University of Tübingen, Tübingen, Germany; 81 German Center for Neurodegenerative Diseases (DZNE), Tübingen, Germany; 82 Department of Psychiatry & Psychology, Mayo Clinic, Rochester, MN USA; 83 Department of Neurodegenerative Disease, Dementia Research Centre, University College London Queen Square Institute of Neurology, London, UK; 84 Department of Pathology and Laboratory Medicine, University of British Columbia, Vancouver, British Columbia Canada

**Keywords:** Neurodegenerative diseases, Genetics research

## Abstract

Atypical frontotemporal lobar degeneration with ubiquitin-positive inclusions (aFTLD-U) is neuropathologically characterized by aggregation of the FET family of proteins and clinically manifests as sporadic young-onset frontotemporal dementia. Here we describe a major risk locus on chr15q14 identified through a genome-wide association study in 59 pathologically confirmed aFTLD-U cases and 3,153 controls (lead single nucleotide polymorphism rs549846383, *P* = 5.85 × 10^−21^, odds ratio 26.7). When combined with data from 28 additional aFTLD-U cases, 3,712 controls and 3,215 individuals with other neurodegenerative diseases and by leveraging in-house and public long-read genome sequencing data from 1,715 individuals, we identified a tandem repeat expansion on the associated haplotypes in an intron of *GOLGA8A*. We found variation in repeat length, motif length, and motif sequence, with long CT-dimer expansions strongly associated with aFTLD-U. Although the functional consequence of this repeat remains unknown, its presence in nearly 60% of aFTLD-U cases points to a fundamental role in disease pathogenesis.

## Abstract (web summary)

Genome-wide analysis coupled with long-read sequencing identifies a repeat expansion in *GOLGA8A* associated with high risk for atypical frontotemporal lobar degeneration with ubiquitin-positive inclusions.

## Main

Frontotemporal dementia (FTD) is a common form of early-onset dementia marked by changes in behavior, language and/or motor function. In individuals 45–64 years of age, the point prevalence varies across studies from 0.02 to 0.22 per 1,000 persons^1,2^. FTD is most often caused by an underlying frontotemporal lobar degeneration (FTLD), with subtypes defined on the basis of the aggregating proteins, with misfolded tau (FTLD-tau) and TAR DNA-binding protein 43 (FTLD-TDP) comprising the largest neuropathological subgroups. The remaining 5–10% of individuals with FTLD show pathological inclusions composed of all three proteins of the FET family (FTLD-FET), that is, fused in sarcoma (FUS), Ewing’s sarcoma protein (EWS) and TATA-binding protein-associated factor 15 protein (TAF15)^3^.

**Fig. 1:**
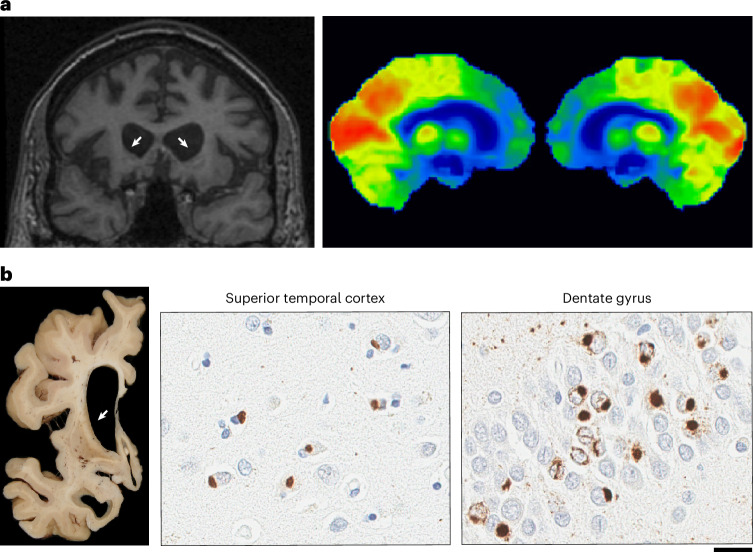
Characteristic neuroradiologic and neuropathologic features of aFTLD-U. **a**, Neuroradiology of aFTLD-U. Bilateral frontal and striatal atrophy (white arrows) is observed with coronal T1-weighted fluid-attenuated inversion recovery magnetic resonance imaging (T1-FLAIR MRI) and bilateral frontal lobe glucose hypometabolism (indicated by blue and green labeled regions) is visible with [^18^F]-fluorodeoxyglucose positron emission tomography imaging of the brain. **b**, Representative image of neuropathology of aFTLD-U. Marked frontal and striatal atrophy is visible macroscopically. Microscopically, pathologic inclusions in aFTLD-U are immunoreactive for FUS and TAF15. Abundant, compact neuronal cytoplasmic inclusions are observed in the superior temporal cortex (anti-FUS antibody; 1:500, 11570-1-AP, Proteintech Group) and dentate gyrus (anti-TAF15 antibody; 1:500, A300-308, Bethyl Laboratories). TAF15-immunoreactive vermiform intranuclear inclusions are regularly observed in the dentate gyrus of aFTLD-U cases. Scale bar, 20 μm.

Genes with a causal role have been identified in FTLD-tau and FTLD-TDP, but not in FTLD-FET. Nearly all individuals with this rare disease subtype lack a family history of a similar illness. FTLD-FET can be further divided into atypical FTLD with ubiquitinated inclusions (aFTLD-U), neuronal intermediate filament inclusion body disease (NIFID) and basophilic inclusion body disease (BIBD) based on differences in the morphology, subcellular localization and anatomic distribution of FET inclusions and other aggregating proteins^4,5^. aFTLD-U is the most common subtype and stands out for its characteristic clinical presentation that typically afflicts individuals in the third to fifth decades with severe behavioral variant FTD (bvFTD), often with pronounced psychiatric disturbance and sparing of language and motor functions^6^. Based on this clinical presentation and the distinct feature of extensive caudate atrophy on magnetic resonance imaging, aFTLD-U can be suspected during a person’s lifetime, but a definitive diagnosis can only be obtained using immunohistochemical analysis at autopsy (Fig. 1).

**Extended Data Fig. 1:**
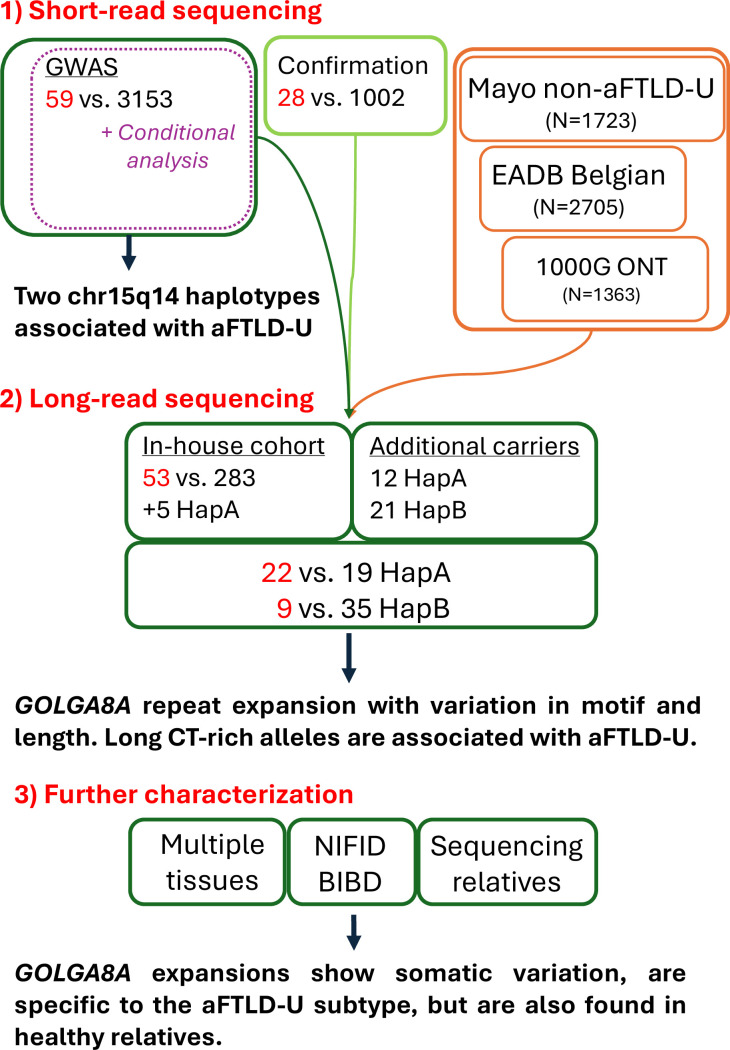
**Schematic overview of the primary analyses and results**.

A schematic of our study is presented in Extended Data Fig. 1. We established an international consortium to assemble a large cohort of aFTLD-U cases. We identified a major associated locus at chr15q14 using a common variant genome-wide association study (GWAS) in 59 aFTLD-U cases and 3,153 controls. We leveraged long-read genome sequencing data from more than 1,700 individuals, which led to the identification of a tandem repeat expansion in an intron of the *GOLGA8A* gene on two associated haplotypes, with extensive variation in repeat length, motif length and motif composition. CT-dimer-rich repeat expansions were strongly associated with aFTLD-U, while CCTT and CCCTCT expansions were also observed in the general population and did not confer aFTLD-U risk.

## Results

### Identification of aFTLD-U associated variants at chr15q14

**Fig. 2:**
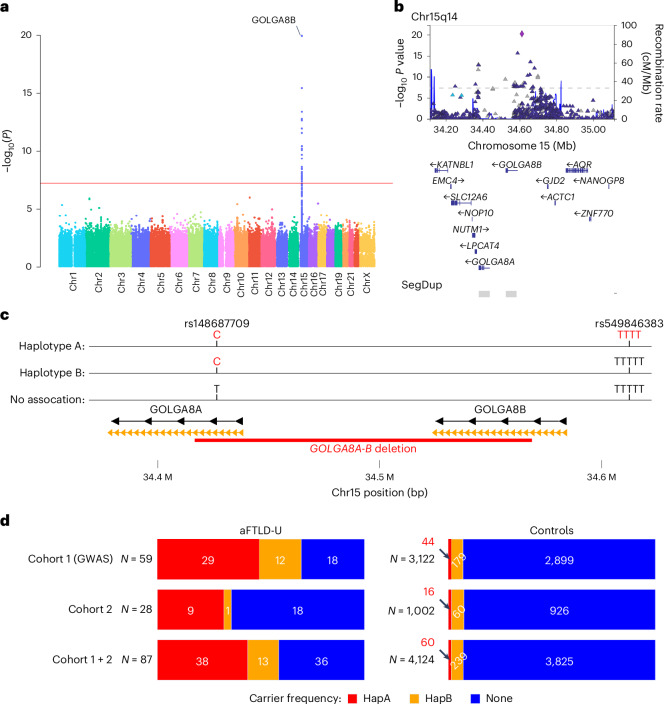
Identification of associated variants and haplotypes. **a**, Manhattan plot showing the result of the GWAS performed using REGENIE for aFTLD-U with a highly significant locus at chr15q14, with 38 genome-wide significant variants. **b**, Visualization of associated variants at chr15q14 based on the GWAS performed using REGENIE, with segmental duplications marked with gray bars. **c**, Schematic visualization of the chr15q14 locus, showing *GOLGA8A* and *GOLGA8B*, the haplotypes we have identified, and the variants identified by GWAS that tag those haplotypes. The segmental duplications with the highest identity are shown in orange, leading to low mappability for short-read sequencing. A frequent deletion overlapping *GOLGA8A* and *GOLGA8B* is shown with a red bar, with genomic coordinates according to the HPRC assemblies^11^ (chr15:34416680–34568563, data accessed through the UCSC genome browser track). **d**, Horizontal bar chart representing frequencies (as shown by color) and absolute number of carriers with associated haplotypes in pathologically confirmed aFTLD-U and control individuals, with individuals with missing genotypes removed.

We performed a single variant GWAS using REGENIE^7^ comparing 59 neuropathologically confirmed aFTLD-U cases and 3,153 controls passing quality control and identified a strongly associated locus at chr15q14 with rs549846383 as the lead variant (*P* = 5.85 × 10^−21^, odds ratio (OR) 26.7, TTTTT > TTTT indel) (Fig. 2a and Supplementary Figs. 1 and 2). This variant was one of 38 genome-wide significant variants at chr15q14 (Fig. 2b), and its minor allele was found in 49.15% (29/59) of aFTLD-U cases compared with only 1.40% (44/3,152) of controls. A similar low frequency was observed in FTLD-TDP cases (7/507; 1.38%)^8^. No additional loci reached genome-wide significance.

The chr15q13-14 region contains pairs of segmental duplications of *GOLGA* genes^9,10^, with rs549846383 telomeric of *GOLGA8B* (Fig. 2c). *GOLGA8A* and *GOLGA8B* are 98.9% identical, which complicates analysis using short-read sequencing because of ambiguous read alignments. In agreement with the HPRC assemblies^11^, we identified copy number variation (CNV) at the *GOLGA8A–GOLGA8B* locus but without disease association (Supplementary Note). A pangene visualization of 472 haplotypes demonstrates the existence of several configurations, with gains, losses and putative gene conversion events^12^ (Supplementary Fig. 3).

We next performed a conditional GWAS by excluding rs549846383 minor-allele carriers, without filtering variants on Hardy–Weinberg equilibrium (HWE) owing to the common *GOLGA8A-B* CNV. The top result from this analysis, comparing 30 aFTLD-U cases with 3,108 controls, highlighted an independent association signal at chr15q14 for rs148687709 (*P* = 3.35 × 10^−5^, OR 4.7) with 40.00% of the remaining cases (*n* = 12/30) and 5.73% (*n* = 178/3,108) of the remaining controls carrying the minor C-allele (Supplementary Figs. 4 and 5). rs148687709 was also strongly associated with aFTLD-U in the original GWAS (*P* = 2.65 × 10^−18^, OR 7.11). In the overall cohort, carriers of rs549846383 form a subset of those with rs148687709, suggesting that rs148687709 tags a haplotype ancestral to the one on which rs549846383 occurred. We refer to the initially discovered haplotype tagged by the minor allele of rs549846383 as haplotype A and refer to the haplotype tagged by the minor allele of rs148687709 (with major allele of rs549846383) as haplotype B (Fig. 2c). Based on gnomAD, the minor alleles of rs549846383 and rs148687709 are most frequently found in non-Finnish European populations (allele frequencies 0.7% and 4.3%, respectively) and especially frequent in the Amish population (allele frequencies 4.7% and 5.5%). As rs148687709 is within the deleted interval of the common CNV, we observed some controls with a heterozygous deletion, carrying the rs549846383 risk allele but without the minor allele of rs148687709, pointing toward a partial deletion of the *GOLGA8A-B* locus on the associated haplotype. The opposite was observed for one aFTLD-U case with a deletion, who appeared homozygous for the rare allele of rs148687709, despite being heterozygous for rs549846383, indicating a deletion of the *GOLGA8A-B* locus on the non-associated haplotype.

Sanger sequencing confirmed the rs549846383 and rs148687709 genotypes observed in our aFTLD-U population and controls and allowed screening of an additional Mayo Clinic control cohort (*n* = 1,002), confirming the low frequency of haplotypes A and B: 16 haplotype A carriers (1.6%) and 60 haplotype B carriers (6.0%). Genotyping of an additional 28 aFTLD-U cases identified 9 haplotype A carriers and 1 haplotype B carrier. Together, in our combined cohort of 87 aFTLD-U cases with DNA available, 38 cases (43.7%) carried haplotype A, 13 cases (14.9%) carried haplotype B, and 36 cases (41.4%) carried neither of the chr15q14 risk haplotypes (Fig. 2d).

### A tandem repeat expansion underlies the association signal

To further characterize the complex chr15q14 locus, we leveraged long-read genome sequencing data from brain tissue from 283 individuals, mostly FTLD-TDP cases and controls, generated as part of ongoing projects. By chance, this cohort already included 2 haplotype A carriers (1 FTLD-TDP case and 1 control) and 14 haplotype B carriers (13 FTLD-TDP cases and 1 control) (Supplementary Table 1). We additionally performed long-read sequencing in brain tissue of 53 aFTLD-U cases (22 haplotype A, 9 haplotype B and 22 carrying neither haplotypes A or B) and 5 non-aFTLD-U individuals carrying haplotype A selected from the FTLD-TDP short-read genome sequencing cohort^8^ (*n* = 2) and the Mayo Clinic control cohort (*n* = 3).

**Fig. 3:**
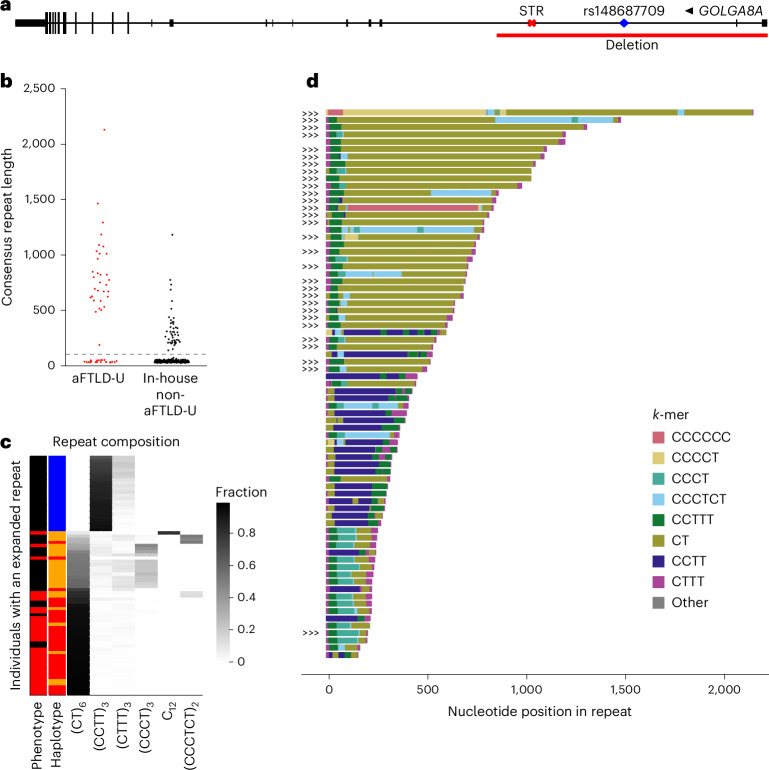
*GOLGA8A* repeat characteristics. **a**, Location of the *GOLGA8A* repeat expansion relative to the *GOLGA8A* MANE transcript (ENST00000359187.5) showing the location of the rs148687709 variant and the deletion. **b**, Length consensus with the length in nucleotides of the repeat consensus sequence of the longest allele for each individual, with a horizontal line at 100 bp (the cutoff for visualizations in **c** and **d**). **c**, Sequence composition plot showing a heatmap of 12-mer motif frequencies, which allows representation of dimer, tetramer and hexamer motifs but does not effectively represent pentamer motifs (observed in one patient). Each row represents a unique individual with an expanded allele (≥100 bp). The first column indicates the phenotype, with aFTLD-U patients in red, the second column indicates the chr15q14 haplotype status of the individual, with haplotype A in red, haplotype B in orange and no associated haplotype indicated in blue. **d**, Plot generated with aSTRonaut showing the repeat sequence for all individuals with an expanded allele (≥100 bp). Colors indicate the observed motifs, and ‘>>>’ annotations preceding the trace mark aFTLD-U patients. A dynamic version of this plot is available at https://wdecoster.github.io/chr15q14/anonymized_aSTRonaut_all.html.

Using the long reads, we confirmed that rs549846383 is in cis with rs148687709. Upon manual inspection of the alignments^13^, we identified an expansion of a short tandem repeat (STR) in an intron of *GOLGA8A* (Fig. 3a) at chr15:34,419,425–34,419,451. After repeat genotyping, we observed repeat length variation in the in-house long-read cohort (*n* = 341), with longer alleles in *cis* with the minor alleles of rs549846383 and rs148687709 and predominantly observed in aFTLD-U cases carrying haplotypes A and B (Fig. 3b). We validated the repeat lengths seen in long-read sequencing by Southern blotting (Supplementary Fig. 6).

We additionally performed a GWAS of aFTLD-U with the length of STRs as continuous predictor variables in the long-read sequencing cohort. A total of 318,299 STR loci passed call-rate filtering, resulting in two genome-wide significant STR loci at chr15q14. We confirmed a strong association for the length of the *GOLGA8A* STR at chr15:34,419,425–34,419,451 (GRCh38) with aFTLD-U (*P* = 1.98 × 10^−13^, OR 17.1). The only other genome-wide significant STR locus was an intergenic repeat polymorphism between *GOLGA8A* and *GOLGA8B* at chr15:34,480,576–34,480,608, which is on average 8 bp longer on the associated haplotypes but without expanded alleles (*P* = 2.02 × 10^−16^, OR 6.2; Supplementary Fig. 7). We further leveraged the long-read sequencing data to genotype all single nucleotide variants (SNVs) and structural variants (SVs) in a 500-kb window around the rs549846383 tagging variant in our cohort, concluding that there are no additional variants that could explain the association signal (Supplementary Note). Using a phylogenetic tree (Methods), we demonstrated that carriers of an associated haplotype cluster separately (Supplementary Fig. 8).

### Substantial variation in repeat motif composition

Encouraged by these findings, we further investigated the associated *GOLGA8A* tandem repeat, which is annotated as an STR with 6.75 copies of a TTTC-motif in GRCh38^14^. However, in our cohort, the analysis of expanded alleles identified expansions of a CT dimer, a CCTT tetramer, a CCCTCT hexamer and CCCCT pentamer motifs. Using a 12-mer heatmap, we observed that CT dimers are found exclusively on haplotypes A and B, occurring at particularly high frequencies in aFTLD-U cases (Fig. 3c). CCTT expansions are observed only in individuals without haplotype A or B, while CCCTCT hexamer expansions are found on haplotypes A and B, but more so in non-aFTLD-U individuals. Representative examples of repeat consensus sequences can be found in Supplementary Table 2. We developed six repeat-primed polymerase chain reaction (PCR) assays with primers against the observed motifs, confirming the repeat sequences observed with long-read sequencing (Supplementary Figs. 9 and 10).

We also observed several flanking motifs, which are variable in length but short (≤20 units), including tetramers (CTTT and CCCT) and a pentamer (CCTTT) (Fig. 3d and Supplementary Fig. 11). Most expanded alleles contained variable lengths of the flanking CCTTT pentamer motif at the 5′ end; more specifically, the reference CTTT units are followed by two to six copies of CCTTT before the sequence transitions into the expanded CT-dimer stretch. All non-aFTLD-U individuals carrying haplotype B showed a short CCCT stretch flanking the 5′ end of the repeat (10–20 units), followed by a short CT stretch.

We also observed mixed repeat compositions. Two aFTLD-U cases carrying haplotype B showed expanded stretches of both CT and CCCTCT. The aFTLD-U case with the CCCCT pentamer motif also has an extended 3′ CT-dimer fragment. We also identified a non-aFTLD-U individual with a 12-mer repeat motif with motif interruptions at the 5′ end and a CT-dimer at the 3′ end of the repeat. Finally, we observed a highly remarkable C_*n*_T-rich allele in a case with haplotype B for which no clear repeat motif could be described. The repeat consensus sequence had up to 62 consecutive Cs, flanked by shorter CT-dimer stretches at the expansion ends. Although the observed long C homopolymer stretches require caution without orthogonal validation, it is noteworthy that this case was the only one with a positive family history of aFTLD-U. Unfortunately, no DNA was available from the affected mother^15^.

Based on these observations, we hypothesized that long expansions predominantly composed of CT dimers drive aFTLD-U risk. In particular, of the seven non-aFTLD-U individuals with haplotype A, one had a CCCTCT hexamer repeat composition, one had a 12-mer repeat and five had CT-rich repeat lengths ranging from only 149 bp up to 1,178 bp (median 433 bp; 71%). By stark contrast, all 22 aFTLD-U cases with haplotype A had long expansions ranging from 489 bp to 2,133 bp (median 760 bp; 100%).

For the 14 non-aFTLD-U individuals with haplotype B, we observed two carriers with a CCCTCT hexamer repeat composition (14.3%) and 12 carriers of a relatively short repeat primarily composed of CT ranging from 187 bp to 235 bp (median 214 bp; 85.7%). By contrast, for seven out of nine aFTLD-U cases carrying haplotype B, we found long expansions predominantly composed of CT-dimer motifs (77.8%), with lengths ranging from 484 bp to 1,245 bp (median 834 bp). The exceptions were the aFTLD-U case with the C_*n*_T-rich sequence described above and one aFTLD-U case carrying haplotype B with a short CT expansion reminiscent of those observed in non-aFTLD-U individuals (presumably with a disease etiology different than chr15q14).

### Characterizing haplotype carriers in additional non-aFTLD-U cohorts

Next, we confirmed the low frequency of the haplotype-tagging variants in non-aFTLD-U by screening additional cohorts of other neurodegenerative disease cases and controls, and we selected an additional 12 haplotype A and 18 haplotype B carriers for detailed long-read sequencing analysis^16–23^ (Supplementary Fig. 12 and Supplementary Note). Among the 12 haplotype A carriers, 2 individuals had no expansion (16.7%) and 3 had a hexamer motif expansion (25%), whereas the other 7 had CT-rich expansions that were relatively short in 2 individuals (137 bp and 159 bp, 16.7%) and longer in the other 5 (325–940 bp, 41.7%). Immunohistochemical analyses confirmed the absence of FUS and TAF15 pathology in non-aFTLD-U individuals with a CT-rich expansion (Supplementary Fig. 13). Among the 18 haplotype B carriers, 16 had CT repeats (88.9%), but the repeat was much shorter than in aFTLD-U cases in all of them, with a mean expansion length of 211 bp and a maximum length of 261 bp. Two haplotype B carriers (11.1%) had a CCCTCT hexamer expansion.

Similar to what we observed for a subset of non-aFTLD-U haplotype A carriers, we expected to find non-aFTLD-U individuals with haplotype B carrying longer CT expansions in rare instances, suggesting that we had not sequenced sufficient haplotypes to observe these. We thus enriched for such carriers by using repeat-primed PCR for the CT motif on all 60 Mayo Clinic controls carrying haplotype B, and selecting 3 individuals with positive signals on one or both sides of the repeat, comparable to what is observed in most aFTLD-U cases with a *GOLGA8A* expansion. Long-read sequencing in these individuals identified longer CT-rich expansions in two (438 bp and 736 bp), with the third control having only a short CT-rich expansion (218 bp). This confirms that a subset of the non-aFTLD-U individuals carrying haplotype A or B may carry long CT-rich repeat expansions comparable to aFTLD-U cases.

### Deriving cutoffs for pathogenic repeat alleles

**Fig. 4:**
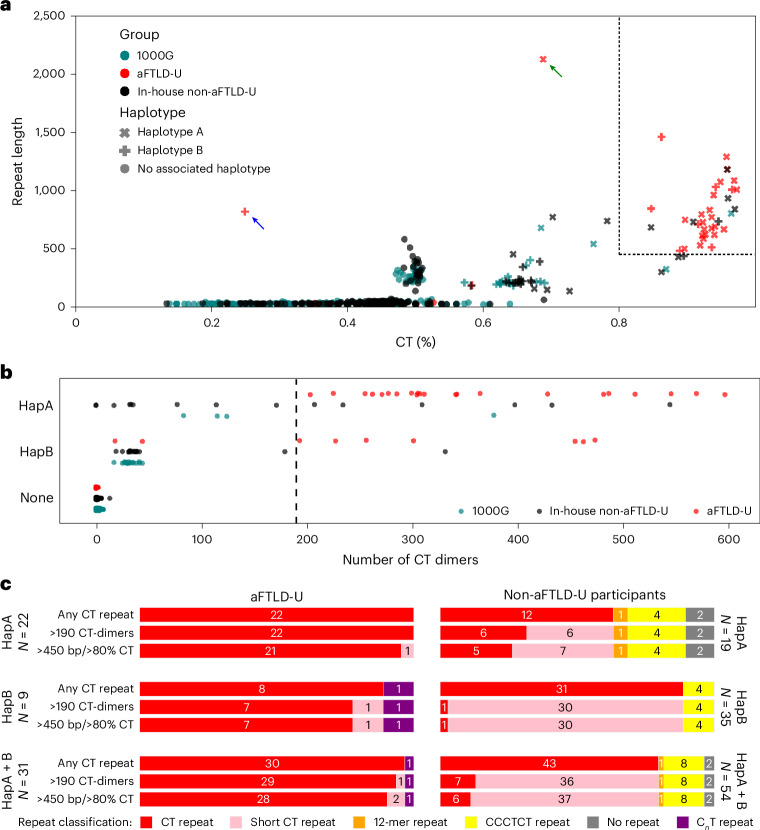
Overview of observed repeats and proposed classifications. **a**, Scatter plot showing the repeat genotype as the percentage CT (*x* axis) and the consensus repeat length (*y* axis, with a minimum of 20 bp), with the cohort as color and haplotype as a symbol. A dotted-line box at 450 bp and 80% CT indicates proposed patient classification cutoffs. A peak of expansions at 50% CT can be seen, corresponding to expansions with the CCTT motif composition. Notable aFTLD-U outliers are indicated with an arrow, that is, the C_*n*_T-rich haplotype B carrier (blue arrow) and the haplotype A carrier with the CCCCT pentamer expansion (green arrow). **b**, Strip plot representing the number of CT dimer units, counted after removing all other CT-containing motifs from the repeat consensus sequence. **c**, Stacked horizontal bar plots of observed repeats and their frequencies (as shown by color coding) and absolute number of carriers in aFTLD-U cases and non-aFTLD-U individuals. Three possible classifications are shown depending on CT-dimer length and percentage CT content. CT-repeats (red) are shown with no length cut-off (‘any CT repeat’), considering only CT repeats >450 bp long and >80% CT, or >190 CT dimer units. CT repeats not matching these criteria are shown in light pink (short CT repeat) in the latter two classifications.

Across all cohorts, long-read sequencing data was available for 19 non-aFTLD-U and 22 aFTLD-U haplotype A carriers and for 35 non-aFTLD-U and 9 aFTLD-U haplotype B carriers. The repeat genotypes of all 1,715 individuals for which long-read sequencing data are available are summarized in Fig. 4a. Repeat characteristics of all haplotype A and B carriers are summarized in Supplementary Table 3.

Based on the current in-house and public data, we propose that a repeat expansion of >450 bp and >80% CT content predicts aFTLD-U cases among haplotype carriers, with a precision of 0.80 (95% confidence interval (CI) 0.64–0.91) and recall of 0.90 (95% CI 0.75–0.97) (Fig. 4a). With an alternative classification, using a threshold of 190 CT-dimer motifs in haplotype carriers (after subtracting other repeat motifs; Methods) (Fig. 4b), we obtain a precision of 0.78 (95% CI 0.62–0.89) and recall of 0.94 (95% CI 0.79–1.00) for the prediction of aFTLD-U. We additionally calculated the association with aFTLD-U for each of the two repeat-based classifiers and compared this with the association with aFTLD-U of the tagging variants, using Fisher’s exact tests in the 1,715 individuals in the long-read cohort. The *P* values are 7.29 × 10^−25^ based on rs549846383 (tagging haplotype A), 2.01 × 10^−29^ based on rs148687709 (tagging haplotype A and B), 5.77 × 10^−40^ for the classification using the double cutoff of >450-bp expansion with >80% CT content, and 4.86 × 10^−41^ for the classification based on expansion alleles with >190 CT-dimer motifs. A schematic overview of the carrier frequency of the repeat based on the two classifications is provided in Fig. 4c. Additional screening of future cohorts is expected to further refine these cutoffs.

### Investigating relatives of haplotype carriers

**Fig. 5:**
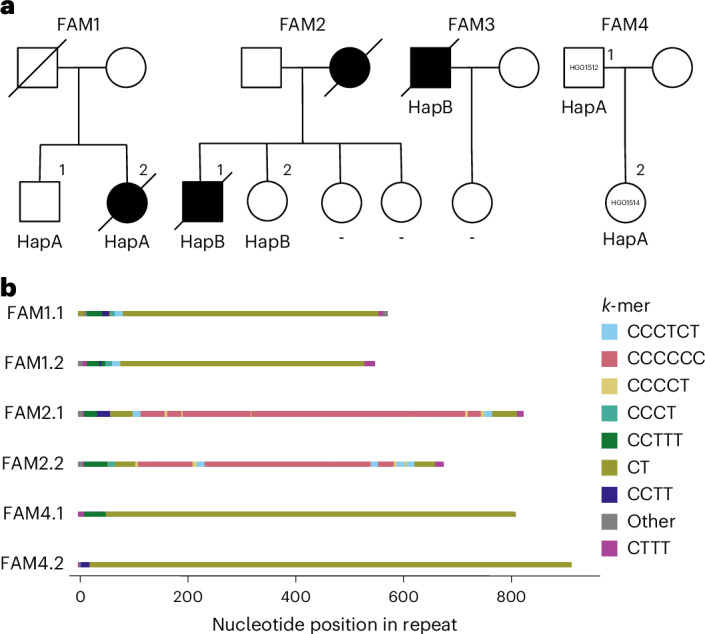
Haplotype carriers and relatives. **a**, Pedigrees of cases and control individuals from the 1000 Genomes Project carrying an expansion for which DNA of relatives could be collected. Cases diagnosed with pathologically confirmed aFTLD-U are indicated with a black shape, and the determined chr15q14 haplotype (A, B or –/none) is shown below the symbol, where DNA was available. Individuals are labeled at the top right with numbers per family. Note that FAM2.2 was lost to follow-up around the age at onset of the affected relative with current disease status unknown, and no DNA was available from the affected mother. **b**, Comparison of the repeat consensus sequence among family members. Individuals are labeled with family ID, followed by the number as indicated on the top right above the symbol in **a**. The consensus sequences for FAM1 family members were generated from LCL-derived DNA; for FAM2, data for the affected brother were from brain-derived DNA, while DNA from the unaffected sister was obtained from blood. Both DNA samples used for FAM4 are extracted from LCLs.

DNA samples could be collected from five unaffected relatives from three aFTLD-U cases carrying the *GOLGA8A* expansion (Fig. 5a). The associated haplotype was present in two unaffected relatives. Long-read sequencing showed that the repeat expansion was similar in size and composition in each family’s affected and unaffected sibling (Fig. 5b). Ultralong nanopore genome sequencing was further performed with DNA extracted from lymphoblastoid cell line (LCL) samples for the sib pair from FAM1, followed by de novo assembly and SV calling without identifying additional variation in the associated locus. From the 1000 Genomes Project cohort (FAM4), we identified one individual (HG01512) with a 804-bp pure CT expansion whose daughter (HG01514) inherited the associated haplotype. Long-read sequencing showed that repeat allele was inherited without substantial further expansion (a 907-bp pure CT expansion; Fig. 5b).

### The *GOLGA8A* repeat shows somatic length variation

**Fig. 6:**
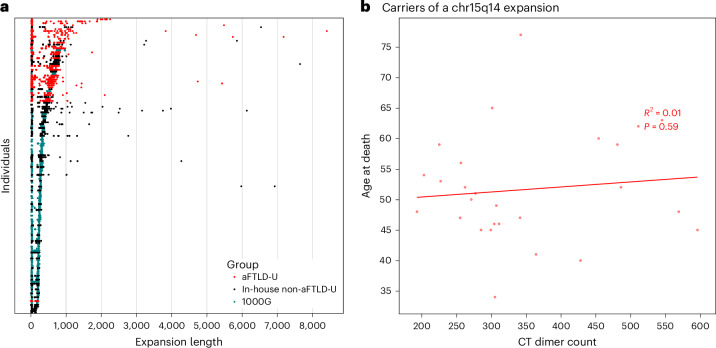
Somatic differences in repeat length. **a**, Strip plot showing, for each horizontal trace, the length per read for all individuals from the in-house and public long-read cohort, including every individual for whom the consensus allele is at least 100 bp. Traces are sorted vertically by the median length of the larger haplotype. Each dot is an individual read and, thus, a separate observation. The frequency of in-house non-aFTLD-U individuals with an expansion does not represent the general population, as we enriched explicitly for those in our sequencing efforts. **b**, Scatter plot showing the correlation of the number of CT dimer units with age at death for aFTLD-U patients for DNA extracted from the frontal cortex. The trendline, the *R*^2^ correlation coefficient and the *P* value were determined using ordinary least-squares regression as implemented in the statsmodels Python module.

We also observed considerable somatic repeat length variation with rare outliers, in agreement with the smear on the Southern blot (Fig. 6a and Supplementary Fig. 6). Visualization of individual reads shows that most of the somatic length differences are in the CT tract (Supplementary Fig. 14). Increased somatic variation, quantified as the standard deviation of repeat length, is observed for longer repeat consensus lengths and not confined to carriers of the associated haplotypes (Supplementary Fig. 15), with the case with the pentamer repeat composition being a notable exception of an exceptionally long expansion with limited somatic length variation. For a small set of cases, we additionally sequenced DNA extracted from other tissues, such as the cerebellum, caudate and occipital cortex, and LCL cultures, again identifying variation in repeat length (Supplementary Fig. 16). We did not observe a correlation between repeat lengths and age in aFTLD-U cases (Fig. 6b).

### Comparison of aFTLD-U cases with and without chr15q14 risk haplotypes

**Fig. 7:**
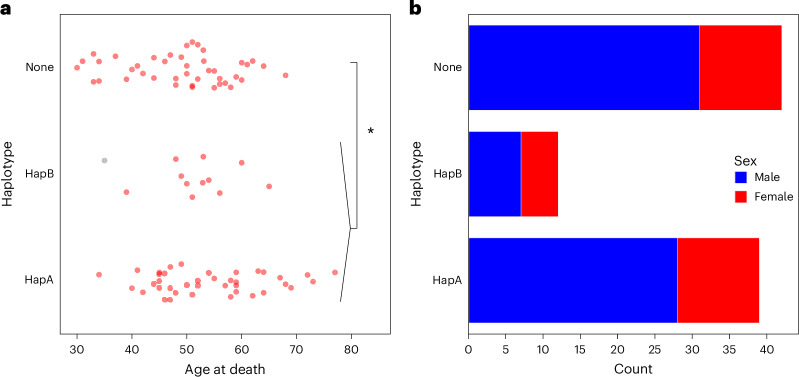
Comparison of demographic features between aFTLD-U cases with and without chr15q14 associated haplotypes. **a**, Comparison of age at death. Two-sided *t*-test between haplotype A or B carriers and those without haplotypes A or B (none): *P* = 0.043. The aFTLD-U case carrying haplotype B but no *GOLGA8A* expansion, as determined by long-read sequencing, is in gray. **b**, Comparison of sex at birth across haplotypes in aFTLD-U cases.

We observed a nominally significant difference in age at death (*P* = 0.043; Fig. 7a) between aFTLD-U cases carrying haplotype A or B and those without association to chr15q14, with a subset of those without the haplotype showing an earlier age at death. In accordance with the distribution in the whole aFTLD-U cohort (Methods), haplotype carriers also show a sex imbalance, with 71% male and 29% female cases (Fig. 7b).

### No role for chr15q14 haplotypes in NIFID and BIBD

We next screened 23 individuals with NIFID and 11 with BIBD for the presence of chr15q14 risk haplotypes, identifying only 3 NIFID with haplotype B. Long-read sequencing was performed for one of these, showing a length (221 bp) and composition of the *GOLGA8A* repeat highly similar to non-aFTLD-U individuals with haplotype B (short CCCT and CT stretches). Insufficient DNA was available for long-read sequencing for the remaining haplotype B carriers.

## Discussion

FTD has a high clinical and neuropathological heterogeneity with three possible disease proteins underlying neurodegeneration^24^. Despite this complexity, genetic FTD risk factors were successfully identified in recent studies owing to adequate patient stratification based on neuropathological classification^25,26^. In the present study, we collected a large cohort of pathologically confirmed aFTLD-U cases for genetic analysis and identified a locus on chr15q14 with 38 variants reaching genome-wide significance. Within this locus, which is characterized by a highly similar and copy-number-variable segmental duplication, we identified two haplotypes associated with aFTLD-U. SNVs tagging these haplotypes were present in nearly 60% of the aFTLD-U cohort, with a notably high OR estimate of 27. Based on long-read sequencing data of >1,700 individuals including aFTLD-U cases, individuals with other neurodegenerative disorders and neurologically healthy controls (Supplementary Table 1), we identified and characterized a STR in an intron of *GOLGA8A*, in *cis* with the haplotype-tagging variants. An increased repeat length and a motif composition with a high CT-dimer content were highly predictive of aFTLD-U and more specific than the tagging variants identified by GWAS.

Interestingly, and distinct from other repeat expansion disorders, the *GOLGA8A* repeat is characterized by a degenerate motif, showing dimer, tetramer, pentamer and hexamer motifs composed of C and T nucleotides, of which some were found to expand and others were flanking the expansion and remained stable in size. We also observed a nearly pure C repeat in the only known inherited case of aFTLD-U, the relevance of which to disease may be clarified in future functional studies. We further observed motif length switches and hybrid compositions within the same repeat allele for which the pathogenic role requires further observations in additional cases and/or controls. Variation in repeat motif composition in disease-associated repeats has been described before, typically involving pentamer repeat motifs, where only specific motifs are pathogenic if expanded^27^. However, the *GOLGA8A* repeat is unparalleled in the variation in repeat length, motif length and motif sequence.

From our collective data, we gathered evidence that long expansions composed of CT dimers are the most likely functional variant underlying disease risk in this locus. First, a detailed analysis of STRs, SVs and SNVs showed no variant that better distinguishes aFTLD-U cases from non-aFTLD-U individuals than the haplotypes A and B tagging variants (rs549846383 and rs148687709). Moreover, *GOLGA8A* repeat expansions of >450 bp and >80% CT content or expansions of >190 CT dimer units showed stronger association with aFTLD-U than the individual haplotype tagging variants. The fact that we observed distinct repeat patterns in the form of CT dimers only on the associated haplotypes, with a clear separation in repeat size between affected and unaffected carriers, further strengthened our findings. That said, based on the available data, we cannot exclude the possibility that other variants on the associated haplotypes contribute to disease risk.

Considerable cell-to-cell somatic variation in terms of repeat length was also observed with most of the variation in the length of the CT-dimer stretches, again pointing to the instability of this specific motif. Somatic variation in tandem repeat length is a well-known feature, especially in the brain. In Huntington’s disease, recent work proposed that expansions in individual neurons may remain innocuous during decades of somatic expansion until they reach a length threshold that confers toxicity and triggers cell death, thus suggesting an active contribution of somatic expansion to disease onset^28^. While somatic expansion could similarly contribute to aFTLD-U, future studies will be required to address this question by linking repeat size and composition in individual neurons to a functional readout. It is also possible that the cells in which the repeat expanded most are no longer present at autopsy. The fact that we did observe expansions in blood-derived LCLs from several individuals indicates that the expansions are not brain specific. Yet, the range of somatic variation across tissues remains to be evaluated.

About 70 repeat expansion diseases are currently described, most leading to neurological or neuromuscular disorders^29,30^. Various mechanisms have been ascribed to these repeat expansions, mainly depending on the location of the repeat relative to an expressed gene, including regulatory effects due to hypermethylation and thus gene silencing, formation of RNA foci often resulting from bidirectional transcription, generation of misfolded proteins for exonic repeats, and repeat-associated non-ATG translation leading to peptide-repeats in multiple reading frames. Importantly, it has been shown that these mutational mechanisms are not mutually exclusive^30^. The aFTLD-U repeat identified in this study is located in an intron of *GOLGA8A*, a gene ubiquitously expressed across organs and tissues, including in the cell types of the brain, with the highest expression in neurons and oligodendrocyte precursor cells (Human Protein Atlas). Transcripts with the expansion could be generated and contribute to disease; however, their identification is challenged by the complex genomic structure of *GOLGA8A* with strong homology to other *GOLGA* gene family members and CNV in the locus. For the same reason, *GOLGA8A* gene expression studies are difficult to interpret. While *GOLGA8A* locus deletions in the general population suggest that loss of *GOLGA8A* expression is not the primary driver of disease, we cannot exclude potential misregulation of the *GOLGA8* gene cluster. Importantly, the specific association with CT-dimer expansions, with no risk associated with CCTT tetramers or CCCTCT hexamers, may point to sequence-specific interactions of the expanded DNA or RNA molecules with other nucleic acids or proteins. Given that this pathological repeat expansion is predominantly composed of a dinucleotide motif, novel mechanisms not previously associated with repeat expansion disorders may also be involved.

Genetic studies in most neurodegenerative diseases have revealed highly penetrant monogenic causes in families and genetic risk factors with weak effect sizes in sporadic cases. While familial gene mutations have occasionally been identified in apparently sporadic cases, the identification of a highly potent risk variant for a disease typically considered sporadic raises the question of why disease segregation is not observed in families. A sporadic appearance would be expected if the repeat expanded de novo in cases, as reported for example for some sporadic neuronal intranuclear inclusion disease cases carrying the GGC repeat in *NOTCH2NLC*^31^. However, for the *GOLGA8A* repeat identified here, it appears the expansions can be inherited, as demonstrated by their presence in non-aFTLD-U individuals, including unaffected relatives of aFTLD-U cases. It is also possible that additional genetic variants are required to develop the disease; however, some degree of familial aggregation would be expected, thus pointing to environmental influences as the most likely contributing factor. Outside the neurodegenerative disease field, there are some notable examples of this, including Moyamoya disease, a rare cerebrovascular disorder, where immune-related responses are thought to interact with a major primary risk variant to induce disease onset^32^. Of particular note is the strong sex bias observed in aFTLD-U cases, with approximately 70% being male, suggesting that sex hormones or intrinsic differences in immune responses between males and females may influence disease penetrance. In future studies, detailed patient histories may reveal possible environmental triggers in aFTLD-U cases.

Finally, repeat expansions at the *GOLGA8A* locus were excluded in 40% of aFTLD-U cases, emphasizing genetic heterogeneity even among neuropathologically indistinguishable phenotypes. As a group, the repeat-negative cases presented with slightly earlier ages at death compared with repeat expansion carriers, with seven cases succumbing before the age of 40, raising the question of what underlies the disease in these individuals. It remains possible that aFTLD-U cases without the chr15q14 risk haplotypes carry comparable expansions at a different genomic locus, similar to what is observed for familial adult myoclonus epilepsy, where TTTCA and TTTTA repeats have been identified in at least six genes^33^. Analogously, a CT-rich repeat expansion anywhere in the genome could function as a prerequisite to developing disease symptoms. In fact, while its origin is unclear, the shared male predominance across 15q14 risk haplotype and nonrisk haplotype carriers could indicate a common underlying disease mechanism. However, unlike familial adult myoclonus epilepsy, the lack of familial aggregation of aFTLD-U combined with the possibility that only one or few cases would have expansions at the same genomic location severely complicates detection of such additional loci. Finally, the *GOLGA8A* repeat was also not associated with NIFID or BIBD, the two other FTLD-FET neuropathological subtypes. These observations are in line with the identification of distinct genetic risk factors for each of the FTLD pathological subtypes, emphasizing the crucial role of investigating phenotypic subsets^26^. Genetic studies focused on gene discovery may become feasible in larger cohorts of NIFID and BIBD cases in the future. Genotyping the haplotype-tagging variants and *GOLGA8A* repeat in individuals with early-onset behavioral symptoms may have diagnostic value for bvFTD and could aid in better classifying pathological subtypes of FTD during life. Further investigation of the downstream consequences of this unusual repeat may provide insight into aFTLD-U disease etiology and identify molecular targets for therapeutic intervention.

## Methods

### FTLD-FET consortium

We established an international consortium to identify and bring together a sufficiently large case population to systematically assess this group of rare disorders. FTLD-FET patients were identified through inquiries at brain banks focused on neurodegenerative disease research and by contacting authors of relevant publications. All patients or their next of kin provided consent to participate in research studies in accordance with the Declaration of Helsinki and local ethics review board standards at each of the participating sites. The ethics committee of the University Hospital Antwerp and the University Antwerp approved the study. Our primary goal was to identify aFTLD-U cases; however, small numbers of NIFID (*n* = 33) and BIBD (*n* = 12) cases were identified and collected during these efforts (Supplementary Table 4).

An experienced neuropathologist from one of the collaborating sites analyzed paraffin-embedded tissue sections for each patient to confirm the neuropathological diagnosis. As our genetic studies primarily focused on aFTLD-U, the patient characterizations were focused on differentiating aFTLD-U from the other FTLD-FET diagnoses. Specifically, aFTLD-U was diagnosed based on the presence of tau- and TDP-43-negative, FUS-positive neuronal cytoplasmic inclusions (NCI) and FUS-positive neuronal intranuclear inclusions (NII). FUS immunostaining was performed at most sites using primary antibodies 11570-1-AP (Proteintech Group) and/or HPA008784 (Sigma Life Sciences), and occasionally A300-302A (Bethyl Laboratories) or aa1-50 (Novus). None of the aFTLD-U cases showed basophilic inclusions (characteristic of BIBD) or other cellular inclusions, such as hyaline conglomerate inclusions (typical of NIFID), on hematoxylin and eosin staining. The diagnosis of aFTLD-U was further supported by the presence of only limited FUS pathology in subcortical regions and limited variability in the morphology of NCIs. In those cases where a differential diagnosis of NIFID was considered, neurofilament or alpha-internexin (AIN) immunohistochemistry was performed to exclude a pathological diagnosis of NIFID. In a minority of aFTLD-U cases, TAF15 immunohistochemistry (A300-308, Bethyl Laboratories) was also performed, confirming TAF15 immunoreactivity (of the inclusions).

So far, our collective efforts have identified 108 aFTLD-U cases from 24 sites, and new cases are being added regularly. The mean age at onset in the full cohort was 44.3 years (median 43, standard deviation 10.4 years, range 21–73 years), with the mean age at death of 51.0 (median 51, standard deviation 10.0 years, range 30–77 years) and a mean disease duration of 6.8 years (median 6, standard deviation 3.4, range 2–19 years). We observed a notable sex imbalance of 34 (31.5%) female and 74 (68.5%) male aFTLD-U cases, which was not observed in NIFID (female *n* = 16, 48.5%; male *n* = 17, 51.5%) or BIBD cases (female *n* = 7, 58%; male *n* = 5, 42%). All cases were self-reported Caucasian except for one aFTLD-U case of Asian ancestry.

Frozen brain tissue from the cerebellum and/or frontal cortex was obtained from 84 aFTLD-U cases, while DNA extracted from blood was available from four additional aFTLD-U cases. For a small subset of aFTLD-U cases, multiple brain regions and LCLs generated by Epstein-Barr virus transformation were available. Only fixed tissue was available for the remaining 20 aFTLD-U cases. A source of DNA was available from 23 out of 33 NIFID cases and 11 out of 12 BIBD cases (Supplementary Table 4).

Inquiry at participating sites also identified a source of DNA (blood or LCL) from five relatives (four siblings and one child) related to three different aFTLD-U cases.

### Additional cohorts

To establish population frequencies of the disease-associated haplotypes A and B and characterize their repeat lengths and sequence composition in non-FTLD-FET and control cohorts, we used several additional populations summarized in Supplementary Tables 1 and 4. These included an in-house cohort of FTLD-TDP cases and controls previously included in long-read sequencing projects, a Mayo Clinic control population including both neuropathologically confirmed normal individuals as well as a clinical cohort of neurologically healthy controls, a cohort of patients with other neurodegenerative diseases (progressive supranuclear palsy, Lewy body dementia and multiple system atrophy) from the Mayo Clinic brain bank (Mayo non-aFTLD-U), Alzheimer’s disease cases and controls from the European Alzheimer’s Disease DNA BioBank (EADB), and individuals from the Oxford Nanopore Technologies (ONT) 1000 Genomes Project^19,20,22^. From the cohort of the ONT 1000 Genomes Project, we identified one repeat expansion carrier who passed on haplotype A to his daughter, for whom only short-read sequencing data was available. We requested an LCL sample from the daughter from the Coriell biobank for long-read sequencing.

The Belgian EADB cohort includes Alzheimer’s disease cases ascertained at the Memory and Neurology Clinics of the BELNEU consortium, and cognitively healthy control individuals who were partners of patients or volunteers from the Belgian community^23^. All control individuals scored >25 on the Montreal Cognitive Assessment test and were negative for subjective memory complaints, neurological or psychiatric antecedents, and family history of neurodegeneration. All participants and/or their legal guardian signed written informed consent forms before inclusion. The study protocols were approved by the ethics committees of the Antwerp University Hospital and the University of Antwerp, and the ethics committees of the participating neurological centers of the BELNEU consortium. Genotyping was performed using the Illumina Infinium Global Screening Array (GSA, GSAsharedCUSTOM_24 + v1.0). Details on quality control, variant calling and imputation have been described in detail by Bellenguez et al.^18^.

### Short-read genome sequencing

DNA samples from 23 aFTLD-U cases and 1,304 neurologically normal controls were sequenced using short-read genome sequencing (phase I) as part of efforts related to the International FTLD-TDP whole-genome sequencing consortium^8,26^. In brief, DNA from 982 control participants from the Mayo Clinic Biobank were sequenced at HudsonAlpha using the standard library preparation protocol using NEBNext DNA Library Prep Master Mix Set for Illumina (New England BioLabs) on Illumina’s HiSeq X. Before analysis, participants from this cohort with possible clinical diagnosis or family history of a neurodegenerative disorder were removed (*n* = 144 removed; *n* = 838 remaining). Whole-genome sequencing for the 23 aFTLD-U cases was performed at the USUHS Sequencing Center, and 322 controls free of neurodegenerative disorders were sequenced at Mayo Clinic Rochester using the TruSeq DNA PCR-Free Library Preparation Kit (Illumina), followed by sequencing on Illumina’s HiSeq X. In a next phase, genome sequencing of 38 newly ascertained aFTLD-U cases (phase II) was performed at Mayo Clinic Rochester using the Nextera DNA Flex Library prep kit followed by sequencing on Illumina NovaSeq. To enhance our study, we further incorporated genomic variant call format (gVCF) files from 2,037 control individuals obtained from the Alzheimer’s Disease Sequencing Project (ADSP). gVCF enables joint genotyping with the existing cohort, as those files provide a comprehensive record of variant calls and reference positions. The gVCF files from ADSP controls were merged with our cohort’s gVCF files using the joint-genotyping approach implemented with the Genome Analysis Toolkit (GATK). By merging these gVCFs, we ensured all our patients and controls were analyzed together, allowing a more robust comparison and reducing batch effects.

For all cases and all controls except those from ADSP, fastq files were processed through the Mayo Genome GPS v4.0 pipeline. Reads were mapped to the human reference sequence (GRCh38 build) using the Burrows-Wheeler Aligner^34^, and local realignment around indels was performed using the GATK. Variant calling was performed using GATK HaplotypeCaller followed by variant recalibration (VQSR) according to the GATK best practices^35^. Variant calling on the final dataset for analysis included the gVCF from 2,037 ADSP control individuals to allow joint genotyping of all cases and controls.

No pathogenic variants in genes linked with neurodegenerative disorders were identified in the aFTLD-U cohort based on genome sequencing and repeat-primed PCR for the *C9orf72* repeat expansion^36^. Mutations in the coding exons of *FUS* and *TAF15* were excluded by Sanger sequencing in patients for whom no genome sequencing data were generated.

### Sample-level quality control

Samples with less than 30× coverage in more than 50% of the genome, call rate below 85%, sex error, or contamination defined by a FREEMIX score above 4 were removed. After joint genotyping of all samples, relatedness was assessed using KING^37^, duplicates were removed and only one individual per family (second-degree relatives or closer) was kept. Individuals with <70% European ancestry based on Admixture analysis were removed^38^. In the aFTLD-U cohort, one case had too low coverage and one Asian case failed ancestry quality control. In total, 59 aFTLD-U cases and 3,153 control individuals passing all quality control measures were included in the analysis (Fig. 2d).

### Variant-level quality control

Genotype calls with genotype quality <20 and/or depth <10 were set to missing, and variants with overall call rate <80% were removed. Gene annotation of variants was performed using ANNOVAR (version2016Feb01).

### Generation of principal components

Before running genetic association analyses, principal component (PC) analysis was performed using a subset of variants meeting the following criteria: minor allele frequency >5% and full-sample HWE *P* > 1 × 10^−5^. Influential regions such as the HLA region were removed, and variants were pruned by linkage disequilibrium with an *r*^2^ threshold of 0.1. We generated PCs, and the top four PCs were included as covariates.

### Genome-wide association analyses

GWAS was performed using REGENIE^7^, including SNVs with minor allele frequency >0.01 in cases or controls and HWE *P* > 1.0 × 10^−6^ in controls. Only variants that passed VQSR filter and with a call rate >90% in both cases and controls were included in the analyses. To remove spurious associations due to potential sequencing batch effects, further filters were applied. Batch effect tests were performed separately for controls (analysis of variance, *P* < 0.01) and cases (Fisher exact test, due to smaller groups), comparing genotype distributions and removing any variant with genotype frequency differences between batches in either cases or controls (*P* < 0.01).

For all remaining 6.9 M variants, the association of genotypes with the case/control status was assessed using REGENIE with allele dosage as the predictor assuming log-additive allele effects. Sex and the first four PCs were included as covariates in the models. We additionally performed a conditional GWAS analysis after removing carriers of the rs549846383 rare allele, applying the same filters described above but without filtering for HWE, testing for association in 7.4 M variants. Variants at chr15q14 were visualized with locuszoom^39^.

A separate cluster of control individuals was identified in the PC plot (Supplementary Fig. 2), and as a sensitivity analysis, we repeated the GWAS while removing those outlier controls, defined as all individuals that are three standard deviations removed on either PC1 and PC2 from the PC center.

### Sanger sequencing genotyping and validations

The rs549846383 and rs148687709 haplotype tagging variants were genotyped using PCR and Sanger sequencing, with primer sequences in Supplementary Table 5. The assay for rs549846383 uses Titanium Taq (Takara Bio), 1 M betaine and 3 min at 95 °C, 32 cycles of 30 s at 95 °C, 30 s at 62 °C and 1 min at 68 °C, with finally 5 min at 68 °C in a Veriti 96-well fast thermal cycler (Applied Biosystems). The assay for rs148687709 is identical, except for a final concentration of 2 M betaine. The results of rs148687709 must be interpreted as tetraploid, as no unique primers could be designed, and the paralogous sequence in *GOLGA8B* will also be amplified (Supplementary Fig. 17). Sanger sequencing results were analyzed using Seqman (DNASTAR) and novoSNP^40^.

### Long-read genome sequencing

Long-read genome sequencing on the PromethION P24 (ONT) was performed for 53 aFTLD-U cases and 5 non-aFTLD-U individuals carrying haplotype A selected from FTLD-TDP short-read genome sequencing and Mayo Clinic controls. For 49 cases, DNA was extracted from the frontal cortex, while DNA from the remaining cases was extracted from the cerebellum. The newly generated dataset was combined with an ongoing genome sequencing initiative of 283 non-aFTLD-U individuals, mostly FTLD-TDP patients and neurologically normal controls. In a second phase, 11 non-aFTLD-U individuals were sequenced, including 8 carrying haplotype A (4 patients with progressive supranuclear palsy, 1 patient with Lewy body dementia, 1 patient with multiple system atrophy and 2 neurologically healthy controls) and 3 neurologically healthy controls carrying haplotype B. An overview of the long-read sequencing cohorts can be found in Supplementary Table 1. We additionally sequenced the genome of one NIFID patient, and sequenced other brain regions (caudate, cerebellum and occipital cortex) and LCLs for selected aFTLD-U cases, as well as LCL- and blood-derived DNA from two unaffected siblings of two aFTLD-U cases. Finally, we requested an LCL sample from HG01514 from the Coriell biobank/NINDS Repository for long-read sequencing.

DNA was extracted from brain tissue using the Nanobind tissue kit (PacBio) and from LCLs with the Qiagen DNA Mini Kit, followed by quality control using the Dropsense (Trinean), Qubit (Thermo Fisher Scientific) and Fragment Analyzer (Agilent) to assess purity, concentration and fragment length. DNA was sheared using the Megaruptor 3 (Hologic, Diagenode) on speed 28–30, followed by removing short fragments with the Short Read Eliminator (PacBio) when considered appropriate. The library prep was generated using the SQK-LSK110 or SQK-LSK114 kit (ONT) according to the manufacturer’s instructions, except for longer incubation times for enzymatic steps, before sequencing on an R9.4.1 or R10.4.1 flow cell for 72 h.

The sequencing data was base called with guppy (for R9 flowcells, v6.7.3) or dorado (for R10 flowcells, v7.1.4, v7.2.13, v7.3.11 and v7.4.13) using the high-accuracy (HAc) base calling model (ONT), including cytosine methylation and hydroxymethylation inference. The data were processed using a snakemake workflow^41^ (github.com/wdecoster/chr15q14). Reads were aligned to the GRCh38 reference genome (GCA_000001405.15_GRCh38_no_alt_analysis_set) with minimap2 (v2.24)^42^, followed by sorting reads by coordinate and conversion to CRAM format with samtools (v1.16.1)^43^. The data quality was assessed with cramino (v0.14.5), as was the concordance with the expected sex based on the normalized read depth of the sex chromosomes^44^. Reads were phased with longshot (v0.4.5)^45^. SVs were called using Sniffles2 (v2.5.3)^46^ and SNVs with Clair3 (v1.0.2)^47^ and Deepvariant^48^, followed by merging variants in gvcf format using GLnexus^49^ and annotation using VEP^50^.

We performed ultralong nanopore sequencing for two participants, a sib pair sharing the haplotype with one affected and one unaffected individual (Fig. 5a, FAM1). DNA was extracted from LCL pellets, following the SQK-ULK114 protocol (ONT) with sequencing on the PromethION and super accuracy base calling (dorado v7.3.11). Obtained data were combined with the standard long-read genome sequencing data (SQK-LSK114), filtered for reads longer than 25 kb using chopper (v0.8.0)^44^ and assembled with hifiasm (v0.24.0-r703) with the –ont option^51^, followed by SV calling with svim-asm (v1.0.3)^52^.

### Tandem repeat analysis

Tandem repeats of interest were genotyped with STRdust (v0.11.7)^53^, either from local files as sequenced in-house or over FTP for the participants from the 1000 Genomes Project resequenced with ONT^19,20,22^. STRdust was used in standard (phased) mode to establish that the repeat expansion is present on the associated haplotype. As read phasing by LongShot was found to be unreliable for this locus, resulting in the omission of a large proportion of the reads from the phased results due to ambiguous alignment and uncertain haplotype assignment, the unphased mode of STRdust was used to obtain the genotypes used in this Article, determining alleles by hierarchical clustering the extracted repeat sequence for each read. STRdust generates a consensus allele by partial overlap alignment as implemented in rust-bio^54^, ignoring length outliers. The observed length variation suggests that the consensus sequence can change substantially due to random sampling of sequenced fragments from the library, especially at low sequencing depth.

The length of all human tandem repeats^55^ was determined using inquiSTR (v0.13.0) (github.com/wdecoster/inquiSTR). We developed STR_regression.R (v1.6) (github.com/wdecoster/inquiSTR/scripts/STR_regression.R) for running association testing of tandem repeat lengths, which can fit generalized linear models using the output of inquiSTR repeat lengths and phenotypic information of multiple samples. STR_regression.R can run both logistic and linear regressions based on binary and continuous phenotypes (and optionally with covariates), and it outputs detailed statistics of repeat length associations. Moreover, it has multiple functionalities, including different repeat length processing modes (considering either mean, minimum or maximum repeat length for a given tandem repeat), various run options (genome-wide, per chromosome and a region of interest based on a chromosomal interval or a list of regions of interest based on a BED file), and it can also take into account provided cutoffs to define expanded alleles of tandem repeats. For this analysis, we compared 52 aFTLD-U cases with 283 non-aFTLD-U individuals, excluding one Asian aFTLD-U case and the five haplotype-A-carrying non-aFTLD-U individuals specifically selected for long-read sequencing. We used the longest allele per individual for all human tandem repeats, with a binary phenotype (aFTLD-U or not), a minimal call rate of 80% and Bonferroni correction for multiple testing.

The repeat composition was assessed using a *k*-mer heatmap, in which all 12-mers were quantified. As the CCCCT pentamer expansion was found in only a single case, the repeat composition in the cohort was quantified and visualized using the least common multiple of 12-mer units to simultaneously represent dimer, tetramer and hexamer motifs, that is, the most commonly observed motifs. VCF files were parsed with cyvcf2 (v0.30.16)^56^, and each 12-mer in the repeat consensus sequences was counted. After counting, all motifs were rotated and represented by the lexicographical first, then collected in a pandas dataframe^57^ before filtering motifs rarely observed, except if highly prevalent in one individual. Visualization was done using Plotly (v5.14.1)^58^. We also used aSTRonaut (v1.0)^53^ to visualize the sequence of the observed repeat motifs per allele (CT, CCTT, CTTT, CCCT, CCCTCT, CCCCT, CCTTT and CCCCCC), replacing motifs by colored dots of the same length, substituting longer motifs first.

We calculated the CT dimer count for each repeat allele by removing all occurrences of other repeat motifs in which CT is a substring (CCCTCT, CCCCT, CCTT, CCCT and CTTT) from the consensus allele and counting the remaining CT units. Precision and recall of the proposed cutoffs (>190 CT dimers or >450 bp repeat and >80% CT) was calculated using scikit-learn (v1.6.1)^59^ with CIs calculated using bootstrapping as implemented in scipy (v1.15.1)^60^.

### Copy number variant analysis

The copy number of the region between *GOLGA8A* and *GOLGA8B* (chr15:34438297–34524132), which is a unique sequence in the human reference genome, was quantified using the coverage obtained from mosdepth (v0.3.8)^61^, normalized to a copy-number-neutral interval (chr15:54033377–56279876) for both short- and long-read genome sequencing data. Visualization was performed in Python using Plotly (v5.14.1)^58^, and statistical analysis was performed for carriers of the deletion allele using a Fisher exact test as implemented in scipy (v1.15.1), comparing the deletion versus normal copy number for aFTLD-U cases against controls^60^.

### Phylogenetic analysis

A phylogenetic tree of haplotypes in the locus of interest (defined as 500 kb surrounding the main tagging variant, chr15:34362469–34862469) was generated using the process described below. First, variants were called with Deepvariant^48^ (v1.8.0) and phased with whatshap^62^ (v2.8). We then selected samples that were fully phased in one phaseblock for the locus of interest using phasius^44^, and removed samples with a copy number suggestive of a deletion or a duplication (removing samples with a normalized copy number below 0.8 or above 1.2). Subsequently, reads were tagged with the haplotype identifier (whatshap haplotag), then splitting the bam file into two haplotypes with samtools split^43^ (v1.13). A consensus in fasta format was generated for each haplotype using samtools consensus, for which then a multisequence alignment was generated using mafft^63^ (v7.526), followed by generating a phylogenetic tree with iqtree^64^ (v2.4.0). The obtained tree was then visualized using ggtree^65^ (v3.14.0).

### Southern blotting

The length of the repeat expansion was confirmed with Southern blotting, using a 437-bp PCR probe, generated from genomic DNA using the PCR DIG Probe Synthesis Kit (Roche) and the following primers: forward: GGACCCTTTAGAGTTGCTTC and reverse: GTATGGAGGGCAGAGTTGTTG (corresponding to chr15:34,420,657–34,421,094). With this configuration, the expected (reference) DNA fragment size is ~4.2 kb. Genomic DNA was extracted from frontal cortex tissue, and 8 μg was digested overnight with Kpn1 and electrophoresed in a 0.8% agarose gel for 6:30 h at 100 V. The DNA was transferred to a positively charged nylon membrane (Roche) by 20-h capillary blotting and then crosslinked by ultraviolet irradiation. Prehybridization in 20 ml DIG EasyHyb solution for 3 h was followed by overnight hybridization at 47.8 °C in a shaking water bath with 30 μl of PCR-labeled probe in 7 ml of DIG EasyHyb. The membrane was washed twice in 2× standard sodium citrate, 0.1% sodium dodecyl sulfate at room temperature for 5 min each, and twice in 0.1× standard sodium citrate, 0.1% sodium dodecyl sulfate at 68 °C for 15 min each. Detection of the hybridized probe DNA was done as described in the DIG System User’s Guide (Roche). CDP-star chemiluminescent substrate was used, and signals were visualized on X-ray film after 30–60 min. The ladders used are the DNA Molecular Weight Marker II with fragments at 23,130, 9,416, 6,557, 4,361, 2,322, 2,027, 564 and 125 bp, and the DNA Molecular Weight Marker VII with fragments at 8,576, 7,427, 6,106, 4,899, 3,639, 2,799, 1,953 and 1,882 bp, and nine smaller bands.

### Repeat-primed PCR

The genomic region on chr15q14 containing the expanded alleles was amplified using a panel of three-primer repeat-primed PCR assays, each with one FAM-labeled primer flanking the repeat, one sequence-specific primer targeting each of the repeat motifs and one booster primer recognizing the tail of the sequence-specific primer to amplify the signal. A total of six primer sets were designed based on observed repeat sequences (Supplementary Table 5), in particular, to determine the presence of CT motifs on the left and right ends of the repeat, CCCTCT motifs on the left and right ends, CCCT motifs on the left, and CCCCT motifs on the left end of the repeat.

The primers are used in equal proportions with amplification using the PrimeSTAR GXL DNA polymerase kit (Takara). Initial denaturation was performed for 2 min at 98 °C, followed by 36 cycles of 10 s at 98 °C, 15 s at 58 °C, and 1 min at 68 °C, with a final extension of 3 min at 68 °C. Fragment lengths were determined with capillary electrophoresis on an ABI3730XL using an internal size standard (LIZ500HD, Thermo Fisher Scientific) and visualized using the in-house developed traci software (v1.1.0) (https://github.com/derijkp/traci).

### Ethics and inclusion statement

As FTLD-FET is a rare disorder, this study was made possible only through a large international collaboration. All colleagues from local sites fulfilling authorship criteria are included in the author list.

### Reporting summary

Further information on research design is available in the Nature Portfolio Reporting Summary linked to this article.

## Online content

Any methods, additional references, Nature Portfolio reporting summaries, source data, extended data, supplementary information, acknowledgements, peer review information; details of author contributions and competing interests; and statements of data and code availability are available at 10.1038/s41588-026-02537-7.

## Supplementary information

Supplementary Information Supplementary Note and Figs. 1–18.

Reporting Summary

Supplementary Tables Supplementary Tables 1–8.

## Data Availability

Individual-level data regarding participants’ phenotype and sex, their *GOLGA8A* repeat characteristics (length, composition, CT dimer count and so on) and the locus copy number are presented in Supplementary Table 3. A dynamic version of the ‘aSTRonaut’ plot 3D is available at https://wdecoster.github.io/chr15q14/anonymized_aSTRonaut_all.html. Summary data on all tested variants of the GWAS analysis are available at https://my.locuszoom.org/gwas/943037/ and in GWAS catalog database under accession code GCST90809297. Short-read whole-genome sequencing data from 23 aFTLD-U cases and 19 controls from phase I were previously deposited in the dbGAP platform as part of the dataset with accession code phs003309 (https://www.ncbi.nlm.nih.gov/projects/gap/cgi-bin/study.cgi?study_id=phs003309.v1.p1). For the 23 aFTLD-U cases, access is restricted: 9 can be for general research use, 1 is for health/medical/biomedical research only and 13 are for ‘disease-specific (neurodegenerative disorders)’ research only. The dbGAP IDs of the patients included in this study are presented in Supplementary Table 6. The 19 controls can also be used for disease-specific (neurodegenerative disorders) research only. Access can be obtained by applying for dbGaP Authorized Access at https://view.ncbi.nlm.nih.gov/dbgap-controlled. The remaining 1,285 controls from phase I are from Mayo Clinic and are not available due to data sharing constraints related to the participants’ consent form. The genetic data for the 38 aFTLD-U cases from phase II are also not part of dbGAP accession phs003309 and not available due to data sharing constraints related to the participants’ consent form. The gVCF genetic data from ADSP used in Phase II are available through a restricted-access policy to not-for-profit organizations; access can be obtained by applying at https://dss.niagads.org/. The long-read sequencing data from HG01514 are available at ENA under the accession ID ERR15094524.

## Code availability

To reproduce the long-read data analysis and figures, all code, in the form of a snakemake workflow, Python scripts and jupyter notebooks, is available via GitHub at https://github.com/wdecoster/chr15q14. The chr15q14 repository is available via Zenodo at 10.5281/zenodo.17965746 (ref. ^66^). STRdust is available via GitHub at https://github.com/wdecoster/STRdust, including the aSTRonaut script, and inquiSTR at https://github.com/wdecoster/inquiSTR, including the STR_regression script.
