# Supplementary material for: A repeat expansion in GOLGA8A is a major risk factor for atypical frontotemporal lobar degeneration with ubiquitin-positive inclusions
Source: Nat Genet. 2026 Mar 12;58(4):726–36. doi: 10.1038/s41588-026-02537-7 (PMC13083237; doi:10.1038/s41588-026-02537-7)
Supplement: Supplementary file 1 — Supplementary Note and Figs. 1–18. [file 41588_2026_2537_MOESM1_ESM.pdf]

nature genetics

Supplementary information https://doi.org/10.1038/s41588-026-02537-7

A repeat expansion in *GOLGA8A* is a major risk factor for atypical frontotemporal lobar degeneration with ubiquitin-positive inclusions

In the format provided by the authors and unedited

Supplementary notes

Copy number variation at the *GOLGA8A-B* locus

While *GOLGA8A* and *GOLGA8B* are highly similar, hampering coverage-based inference of copy number, the sequence between these genes is unique in the reference genome. After counting reads and normalization (see Methods) we were able to identify deletion and duplication alleles of the unique sequence between *GOLGA8A* and *GOLGA8B* with 80.9% of the cohort having a diploid copy number, 17.2% having a heterozygous deletion, 1.0% having a heterozygous duplication, and 0.9% having a homozygous deletion (**Supplementary Figure 18**). Only diploid copy number (N=49) and heterozygous deletions (N=10) were observed in the short read data of aFTLD-U patients. Statistically testing for disease association of the deletion allele with aFTLD-U, by comparing against the frequency of the deletion in controls using a Fisher's exact test, indicated there was no difference in frequency (p-value = 1.0).

Comprehensive assessment of variation in the associated locus

To catalog additional functional variants beyond STRs that may explain the association signal and could have been missed with the initial short-read sequencing association study, we conducted a comprehensive evaluation of all types of variants in the associated locus, arbitrarily defined as a 500kb interval around the rs549846383 tagging variant (chr15:34362469-34862469) using our long-read sequencing data. This analysis was performed in a cohort of aFTLD-U patients (N=52) and non-aFTLD-U subjects (N=283), omitting the small set of specifically selected non-aFTLD-U individuals carrying haplotype A to guarantee an accurate representation of the general control population. Variants found in fewer than five aFTLD-U patients were omitted. We counted the number of patients and controls for every variant. Next, we performed Fisher's exact test to test for association with the phenotype and compared these with the p-values obtained for the tagging variants.

Structural variants (defined as fragments ≥ 50bp with a change in location or copy number, **Supplementary Table 7**) were genotyped with Sniffles2 in population mode. Since STR length variation was already evaluated as part of the repeat-length GWAS analysis, we omitted any repeat length variation from this analysis, as Sniffles typically splits repeat length polymorphisms across multiple VCF records. Sniffles also failed to genotype a 400bp insertion upstream of *GOLGA8A* (chr15:34454183), which was thus genotyped with custom

code. In total, 10 SVs were identified and evaluated, including some insertions or deletions that were commonly found on the associated haplotypes. This also included two structural variants found in every individual in the entire cohort, indicating positions where the reference genome is either incorrect or carries a rare allele. None of the 10 variants was found to be more strongly associated with aFTLD-U than the haplotype tagging variants (most significant p-value 4.26×10^{-7} in SV analysis as compared to 1.69×10^{-18} for haplotype tagging variant). Moreover, all SVs were present in more individual non-aFTLD-U subjects than the haplotype tagging variants.

Next, all SNVs were genotyped in the 500kb locus using Deepvariant, with gvcf files merged with GLnexus, resulting in 2091 variants seen in at least five patients (**Supplementary Table 8**). The most significantly associated variants were again the two tagging variants observed in the short-read GWAS analysis (rs549846383 and rs148687709). We annotated all variants with VEP to identify variants with a potential pathogenic impact, and out of the 2091 variants, only 18 were predicted to be missense variants. We performed an assessment of the LD (r^2) of all variants relative to both tagging variants. Finally, using 20 long-read genomes for which the SNVs could be phased in one contiguous phase block across the 500kb interval, we determined the percentage of instances where the variant was physically observed on the same haplotype as rs549846383. Altogether, this demonstrated that there were no SNVs missed in the short-read GWAS with a stronger association than rs549846383 and rs148687709, and that all other SNVs that almost perfectly segregated with rs549846383 were (much) more frequent in controls than rs549846383.

The above findings, combined with the fact that the intergenic repeat polymorphism at chr15:34,480,576-34,480,608 identified in the repeat-length GWAS, appears to tag the disease-associated haplotype, but does not differentiate between aFTLD-U and non-aFTLD-U patients in terms of repeat length (**Supplementary Figure 8**), leads us to conclude that the *GOLGA8A* STR is most likely to functionally explain the association signal. Additional support for this conclusion based on repeat length, repeat composition, and statistical analysis focused on pathogenic cut-offs is provided in the main manuscript.

Screening additional cohorts for haplotype carriers

First, available short-read genome sequencing data from patients with tauopathies and a-synucleinopathies (Progressive supranuclear palsy (PSP), Lewy body dementia (LBD) and Multiple system atrophy (MSA); N=1,723) from the Mayo Clinic Brain Bank was queried for the haplotype-tagging variants showing 22 heterozygous carriers of haplotype A (1.28%) and

61 carriers of haplotype B (3.54%)[2,3]. Out of the haplotype A carriers we randomly selected six individuals for long-read sequencing. Second, imputed genotypes for rs549846383 were available for individuals from the European Alzheimer's Disease DNA BioBank (EADB) Belgian cohort (1,474 controls and 1,230 Alzheimer Disease patients)[4]. This identified an equal proportion of heterozygous haplotype A carriers in the controls (18; 1.22%) and Alzheimer's Disease patients (15; 1.22%). Two controls with haplotype A were randomly selected for long-read sequencing. We additionally analyzed 1,363 individuals from the long-read genome sequencing initiatives in the 1000 Genomes Project[5–8], identifying four haplotype A carriers (0.34%) and 18 haplotype B carriers (0.85%). For the latter cohort, long-read genomes were generated on LCL-derived DNA, which, in theory, could lead to within-culture variability resulting from cell passaging, yet we did not see evidence for this. Combined, 12 haplotype A and 18 haplotype B carriers were selected/identified for detailed long-read sequencing analysis.

Supplementary Figures

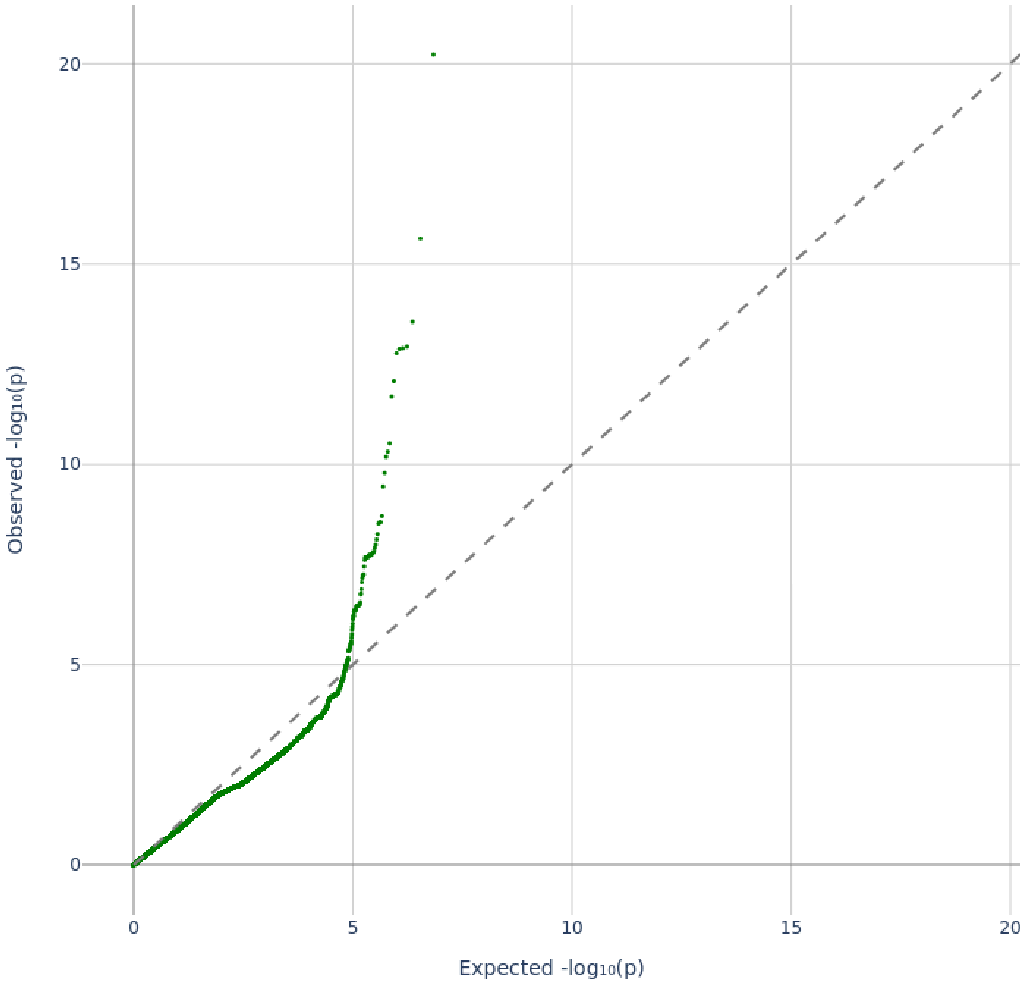

Supplementary Figure 1: QQ plot for the GWAS analysis, with lambda=0.887.

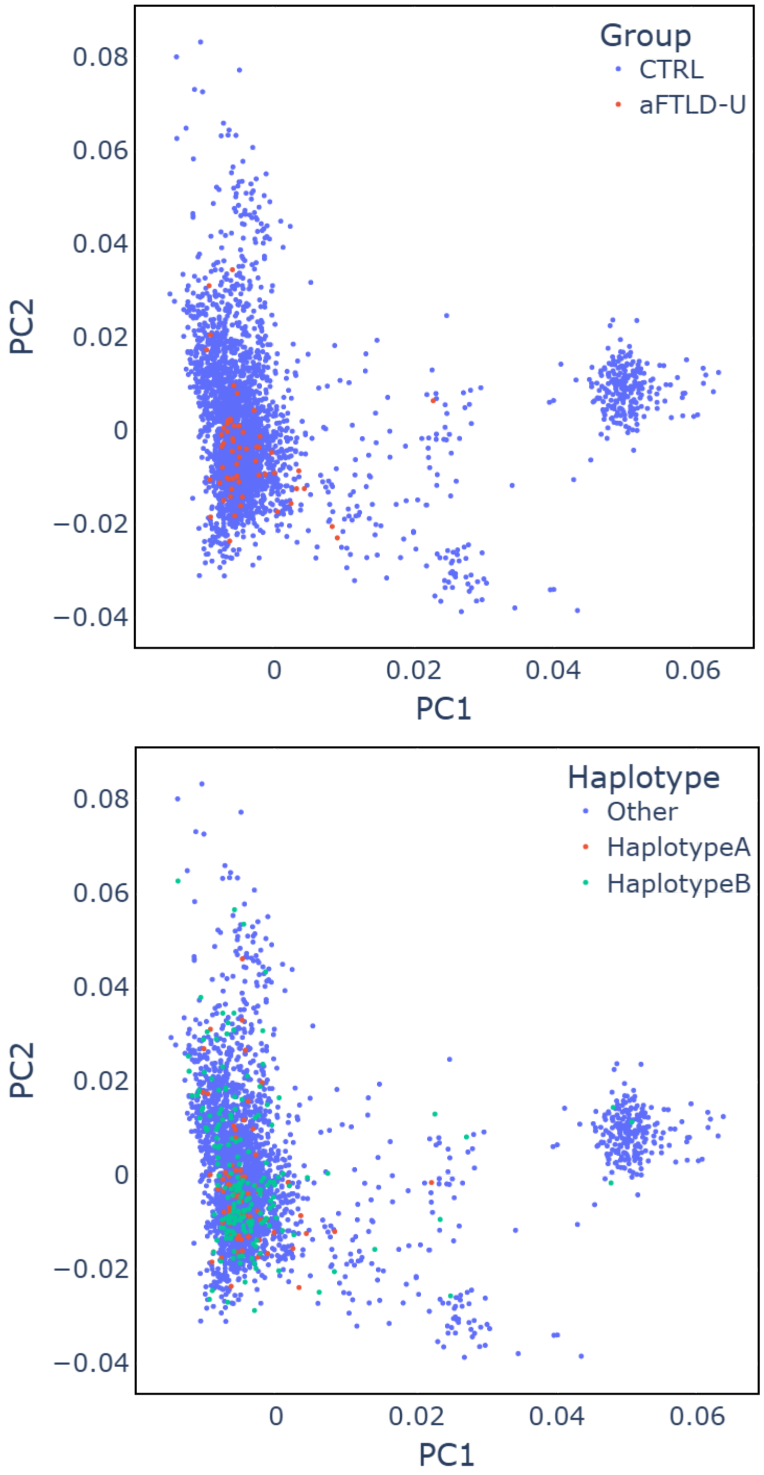

Supplementary Figure 2: PC plot for the GWAS showing disease status (top) and haplotype carrier status (bottom). Removal of control individuals who appeared as a separate cluster only improved the association signal (rs549846383: p-value = 3.70x10-23, OR = 31.40).

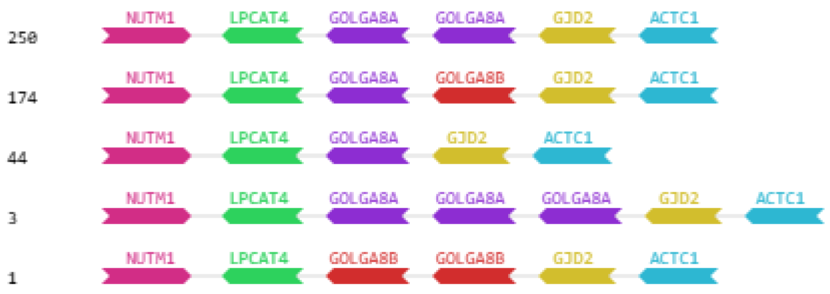

Supplementary Figure 3: pangenome visualization from 472 haplotypes concerning the *GOLGA8A* and *GOLGA8B* genes, identifying gains and losses of GOLGA8A or GOLGA8B and potential gene conversion events resulting in tandem organization of two or more copies of the same gene. (http://pangene.liheng.org/view?graph=human472-1.1a2&gene=GOLGA8A%2CGOLGA8B&step=2&ori=)

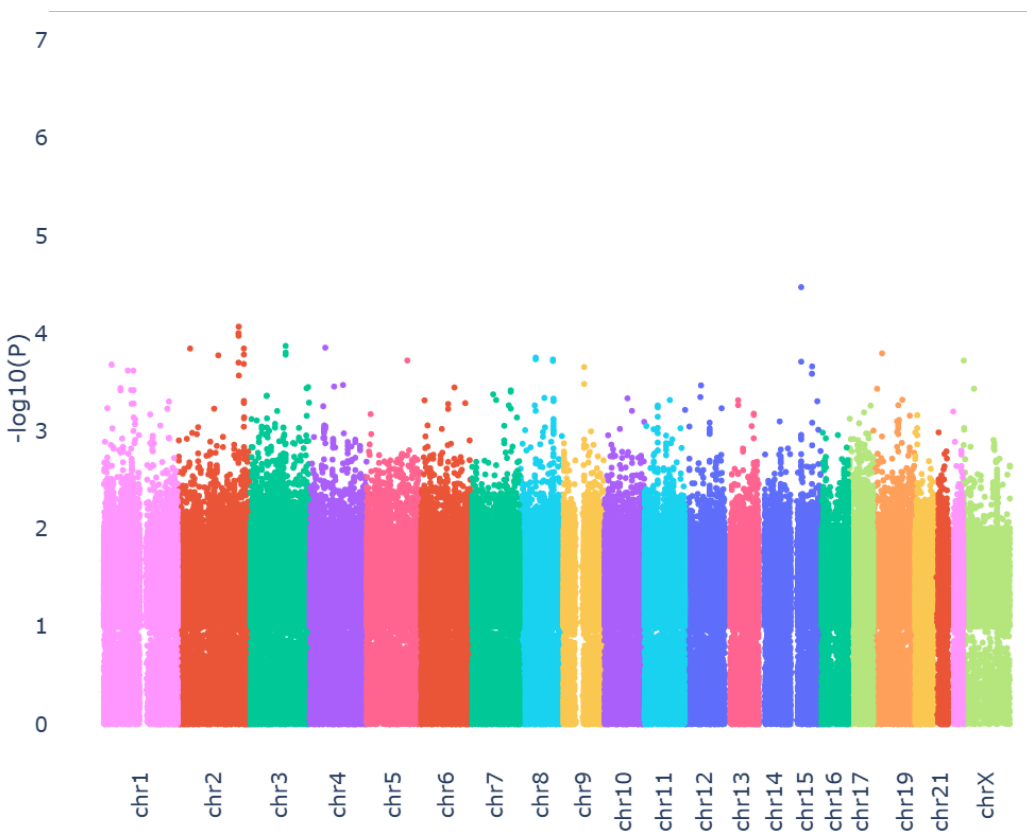

Supplementary Figure 4: Manhattan plot from the conditional GWAS analysis (rs549846383 minor-allele carriers excluded).

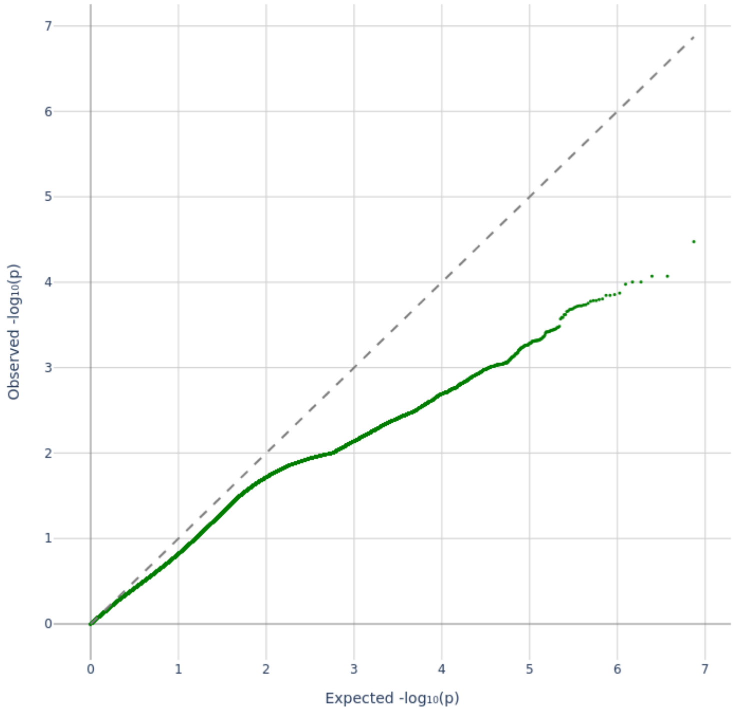

Supplementary Figure 5: QQ plot from the conditional GWAS analysis (rs549846383 minor-allele carriers excluded).

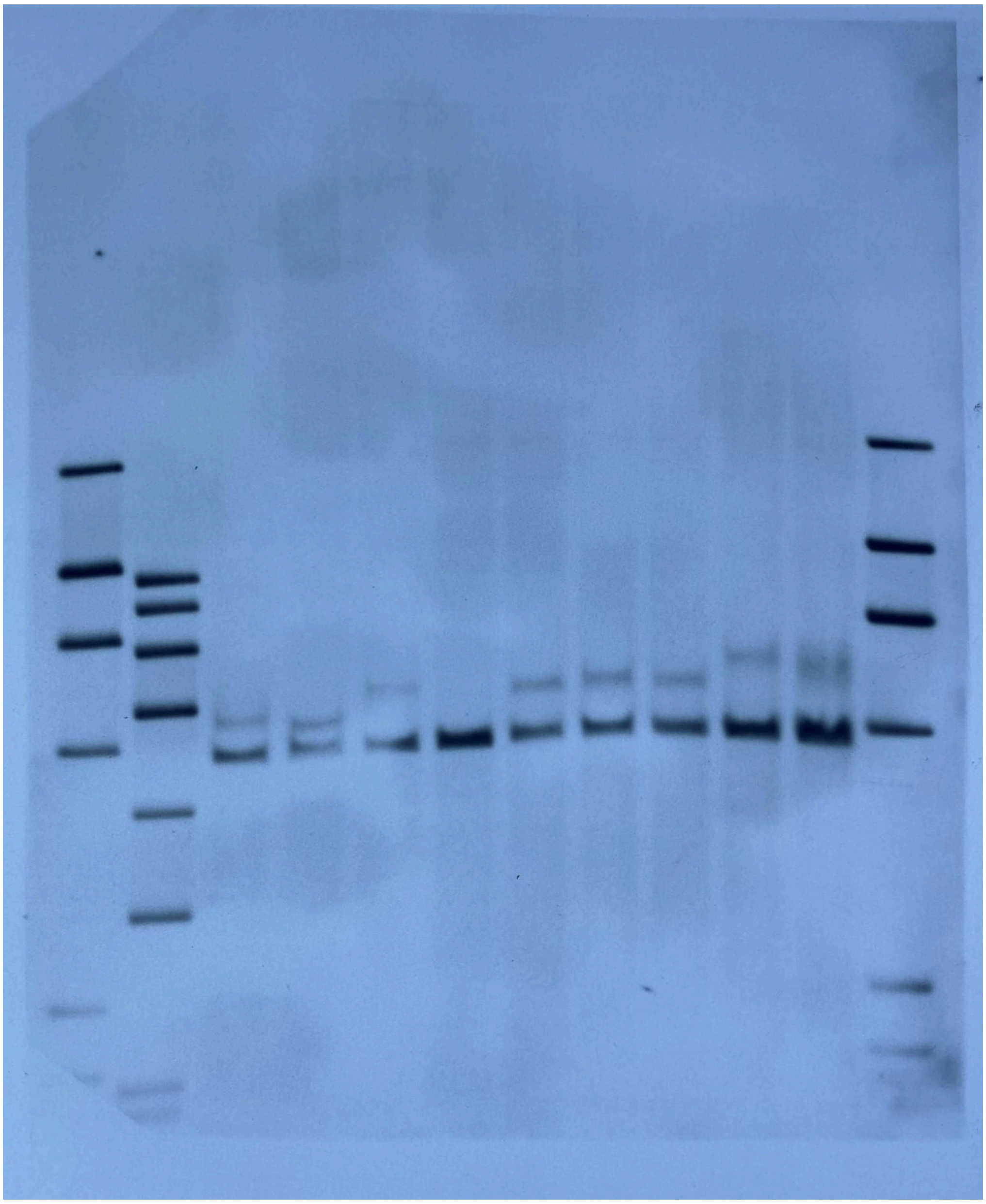

Supplementary Figure 6: Southern blot using DNA extracted from frontal cortex tissue to validate the observed repeat lengths (details see Methods). From left to right: DNA Molecular Weight Marker II (ladder sizes, in basepairs: 23130, 9416, 6557, 4361, 2322, 2027, 564, 125), DNA Molecular Weight Marker VII (ladder sizes, in basepairs: 8576, 7427, 6106, 4899, 3639, 2799, 1953, 1882), three different non-aFTLD-U subjects carrying haplotype A, one non-aFTLD-U subject without risk haplotype A or B, five different aFTLD-U patients with haplotype A and another DNA Molecular Weight Marker II. All tissue samples were from the Mayo Clinic Brain Bank.

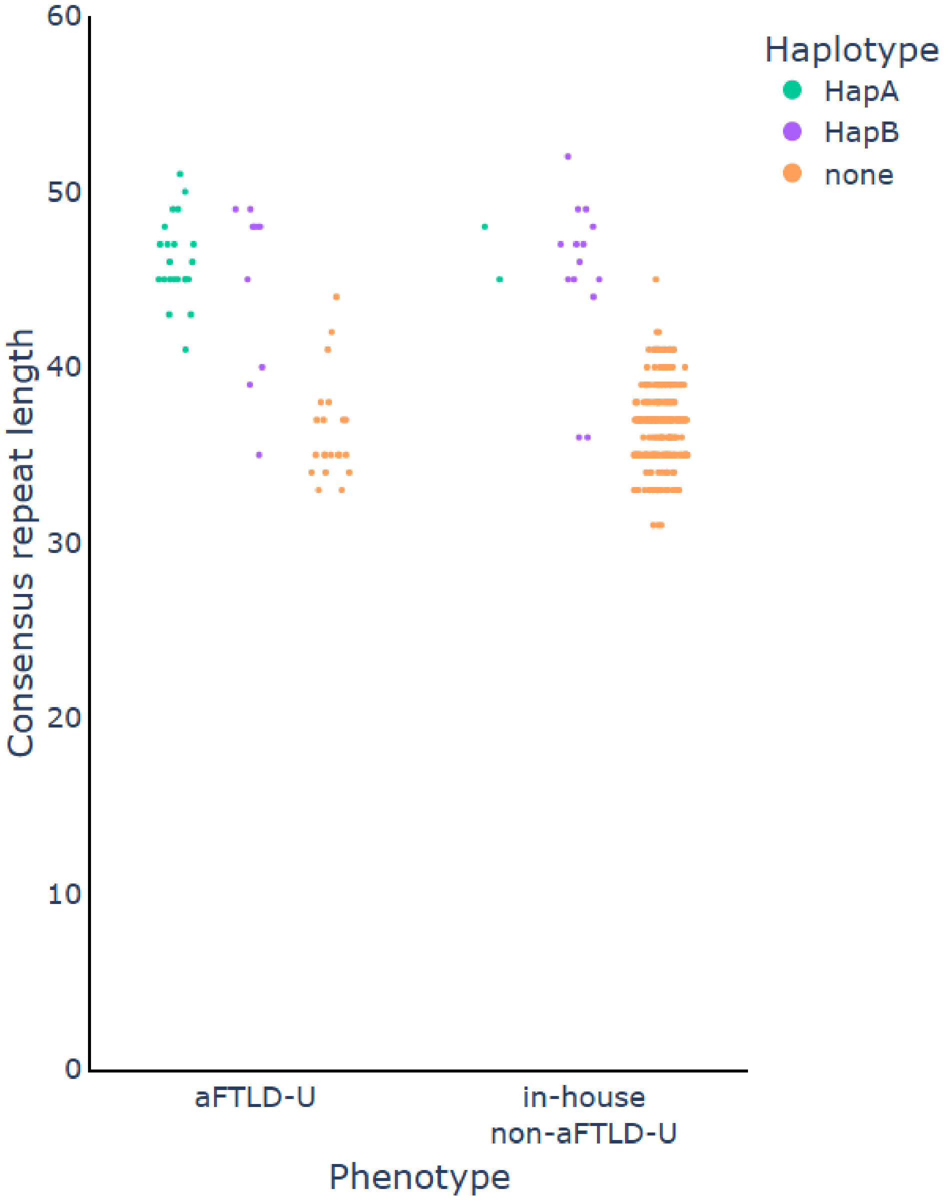

Supplementary Figure 7: Length of the associated intergenic tandem repeat at chr15:34480576-34480608, genotyped using STRdust in the ONT cohort (excluding intentionally included haplotype-A-carrying non-aFTLD-U subjects), with the y-axis indicating the full repeat length in nucleotides (including the length on the reference genome, 33bp). The repeat is an imperfect GT dimer repeat. The mean length of individuals without a haplotype is 37bp, the mean length for Haplotype A or B carriers is 45.6bp.

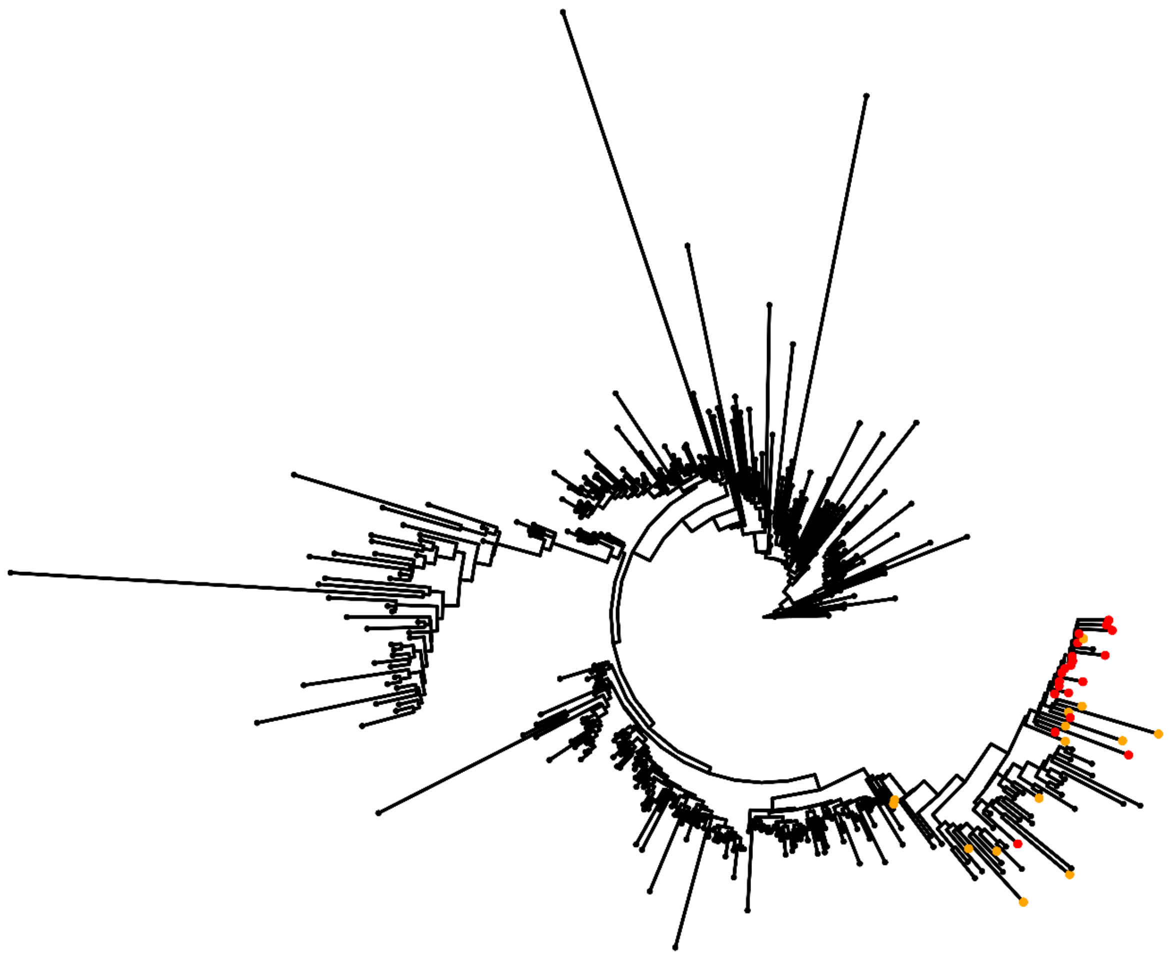

Supplementary Figure 8: phylogenetic analysis of haplotypes in the locus of interest (chr15:34362469-34862469), showing associated haplotype A in red and associated haplotype B in orange (see Methods), including 426 haplotypes from 213 individuals.

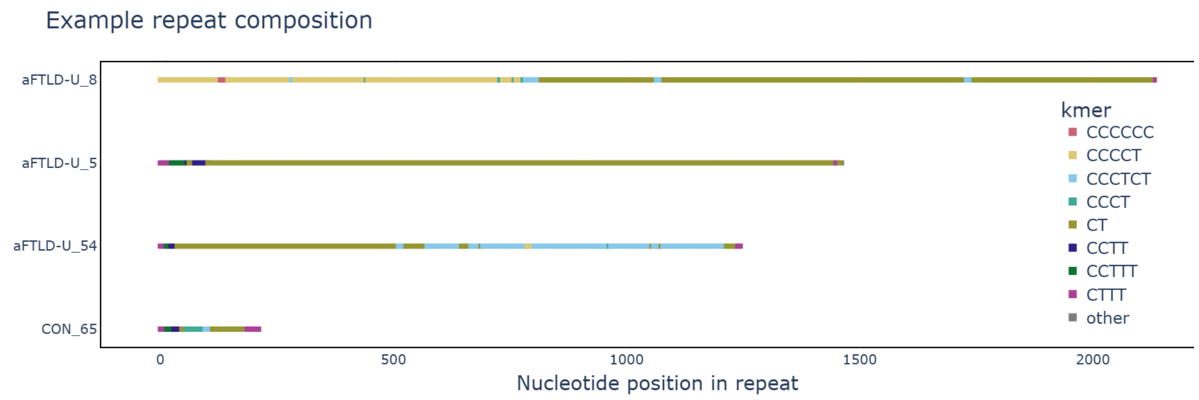

Supplementary Figure 9: aSTRonaut plot showing the repeat structure of selected samples to illustrate the repeat-primed PCR results (see also **Supplementary Figure 8** with sample labeling in reference to **Supplementary Table 5**).

Repeat-primed PCR ‘CT-left’ assay results

aFTLD-U_8

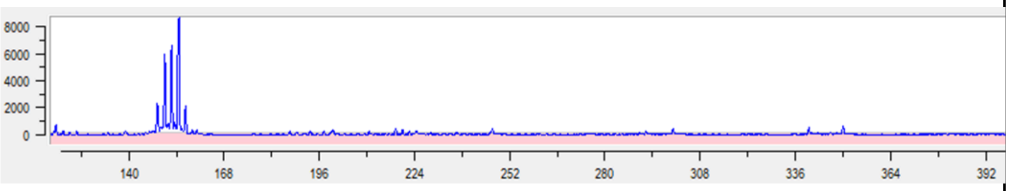

aFTLD-U_5

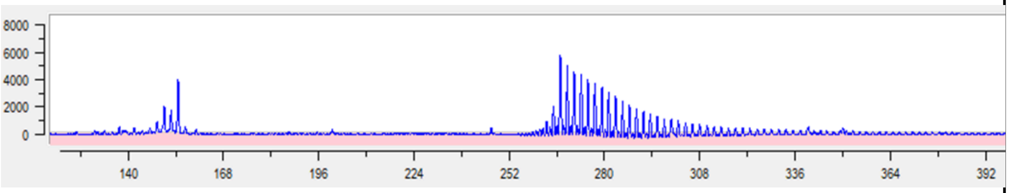

aFTLD-U_54

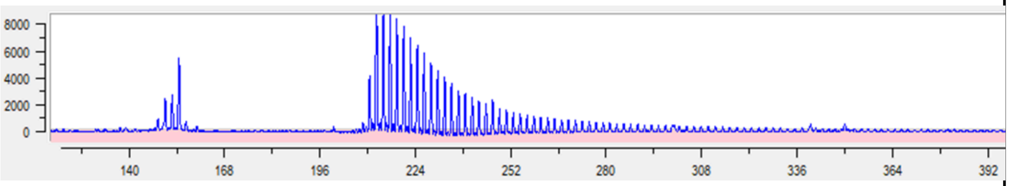

CON_65

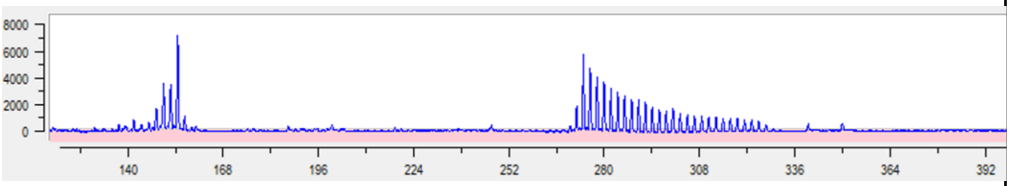

Repeat-primed PCR ‘CT-right assay results

aFTLD-U_8

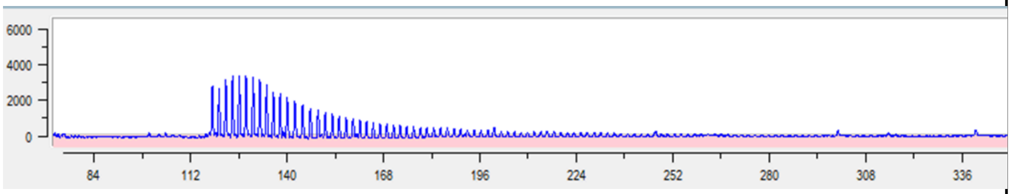

aFTLD-U_5

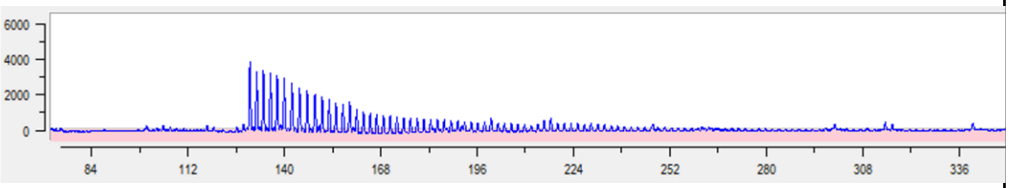

aFTLD-U_54

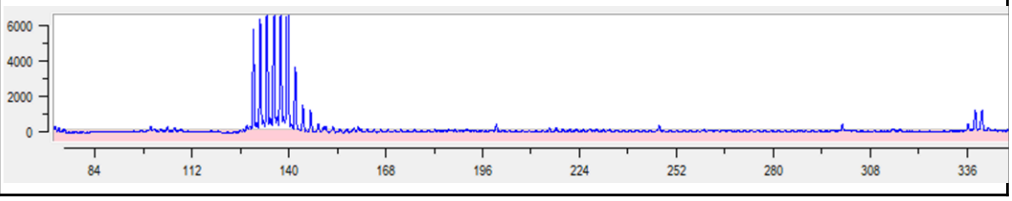

CON_65	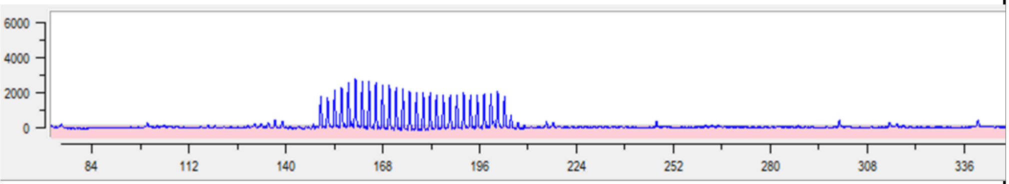
**Repeat-primed PCR ‘CCCTCT-left assay results**	
aFTLD-U_8	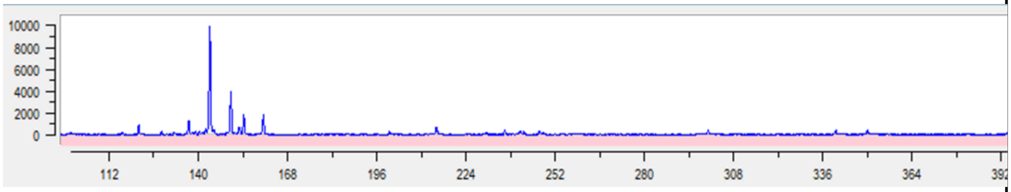
aFTLD-U_5	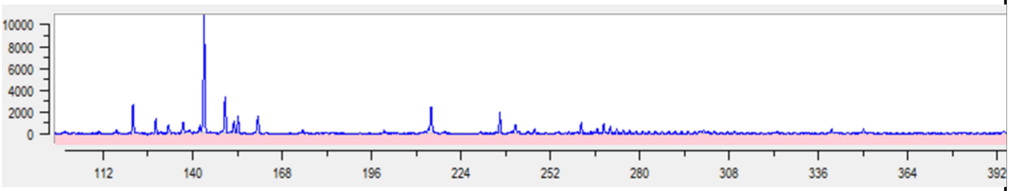
aFTLD-U_54	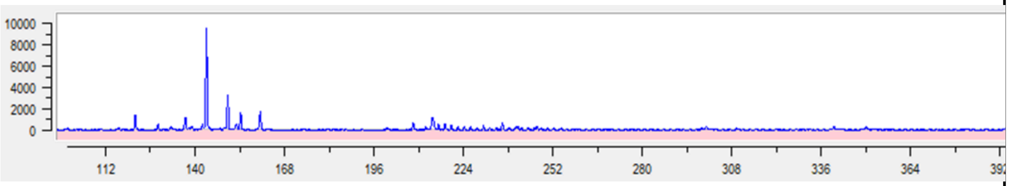
CON_65	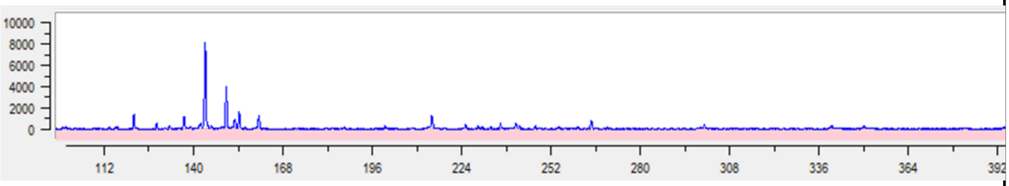
**Repeat-primed PCR ‘CCCTCT-right assay results**	
aFTLD-U_8	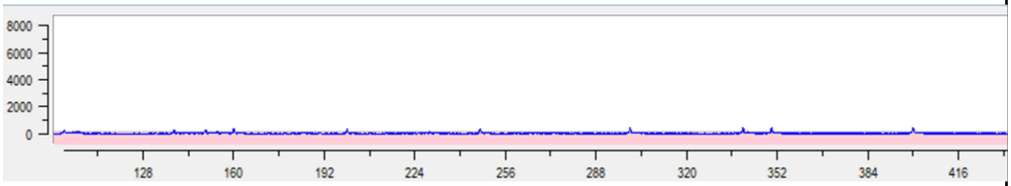
aFTLD-U_5	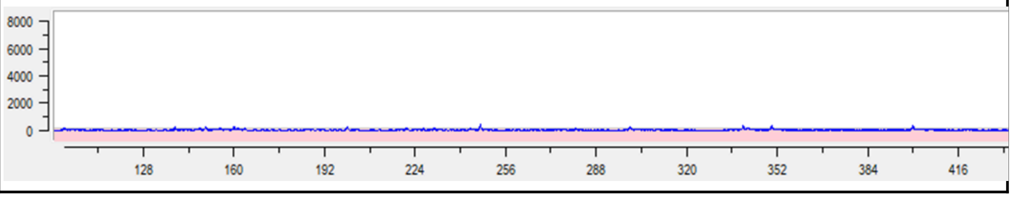

aFTLD-U_54

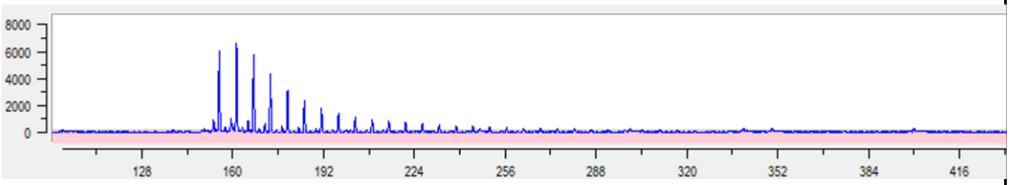

CON_65

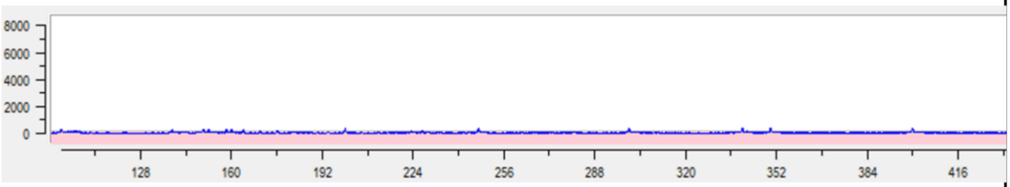

Repeat-primed PCR ‘CCCT-left assay results

aFTLD-U_8

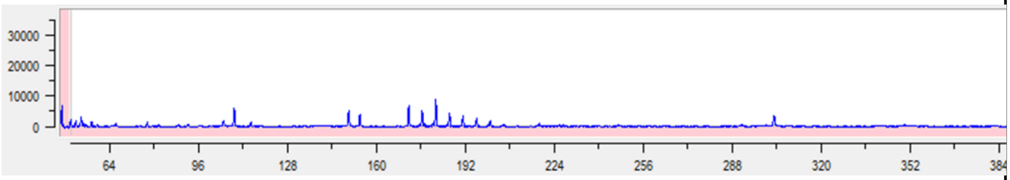

aFTLD-U_5

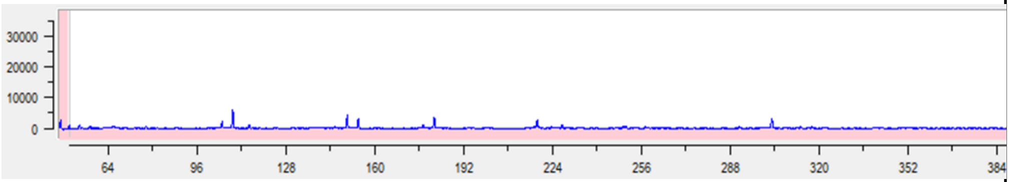

aFTLD-U_54

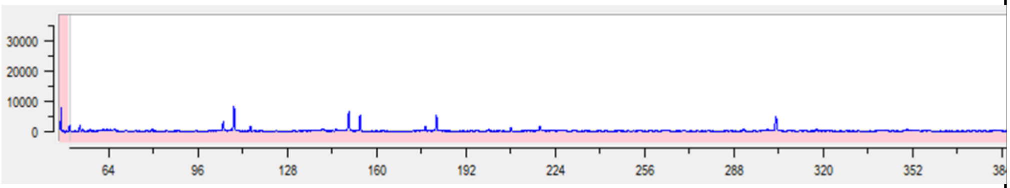

CON_65

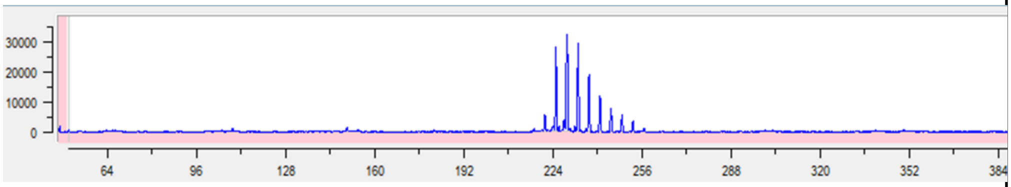

Repeat-primed PCR ‘CCCCT-left assay results

aFTLD-U_8

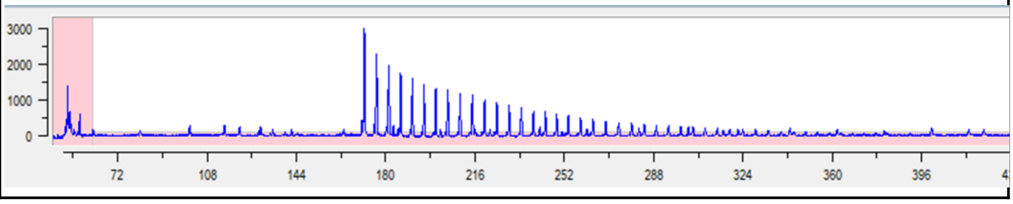

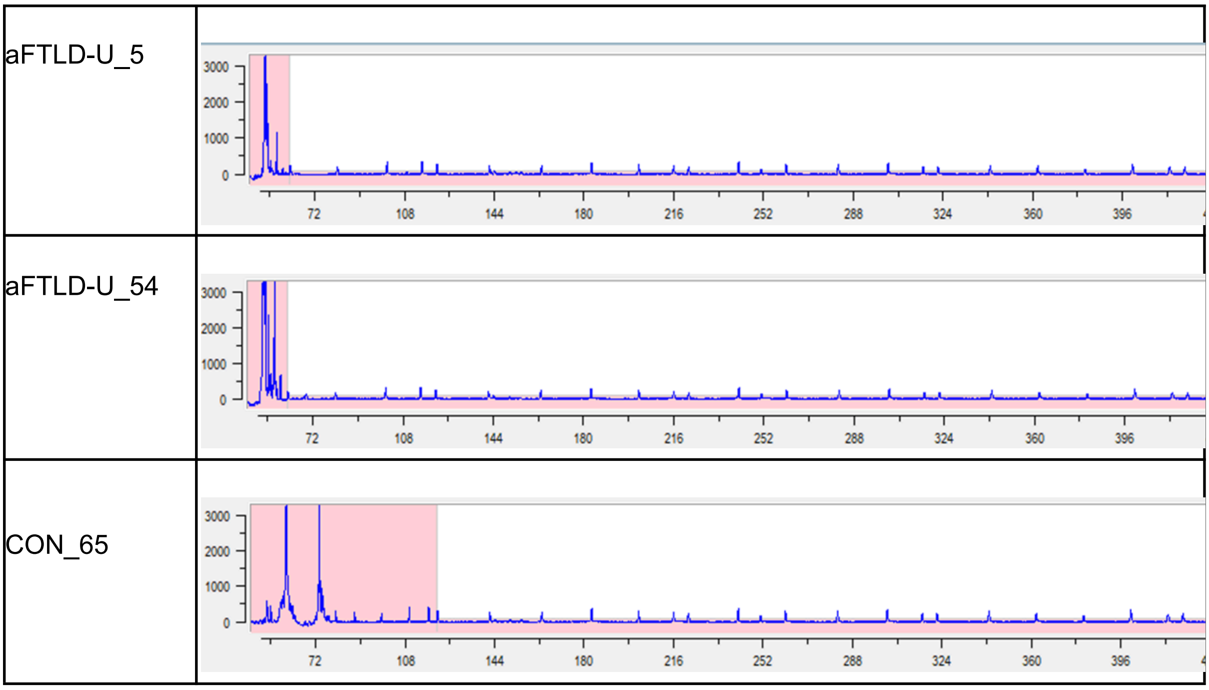

Supplementary Figure 10: Examples of repeat-primed PCR assays corresponding to the individuals in **Supplementary Figure 9**. aFTLD-U_8 shows a characteristic stutter pattern indicative of a repeat-expansion using the CT-right assay but fails to show amplification of the CT-left assay due to the presence of a different motif, e.g. a CCCCT-expansion on this side. aFTLD-U_5 shows characteristic stutter patterns for the CT-left and CT-right assays and is negative for all other assays. aFTLD-U_54 shows the characteristic stutter-pattern using the CT-left assay, and a stutter pattern using the CCCTCT-right assay. Note that there is also amplification of a small stable fragment using the CT-right assay most likely due to the presence of small CT-dimer stretches within the CCCTCT repeat as also suggested by the aSTRonaut plot. Finally, CON_65 carries the typical haplotype B seen in non-aFTLD-U subjects with a short motif of CCCT-tetramers and CT-dimers. Due to the small total size of the repeat both the CT-left and CT-right assays show amplification; however, the pattern suggests a smaller (and largely stable size) of the CT-expansion. Positive amplification was also seen for the CCCT-left assay whereas all other primer sets were negative.

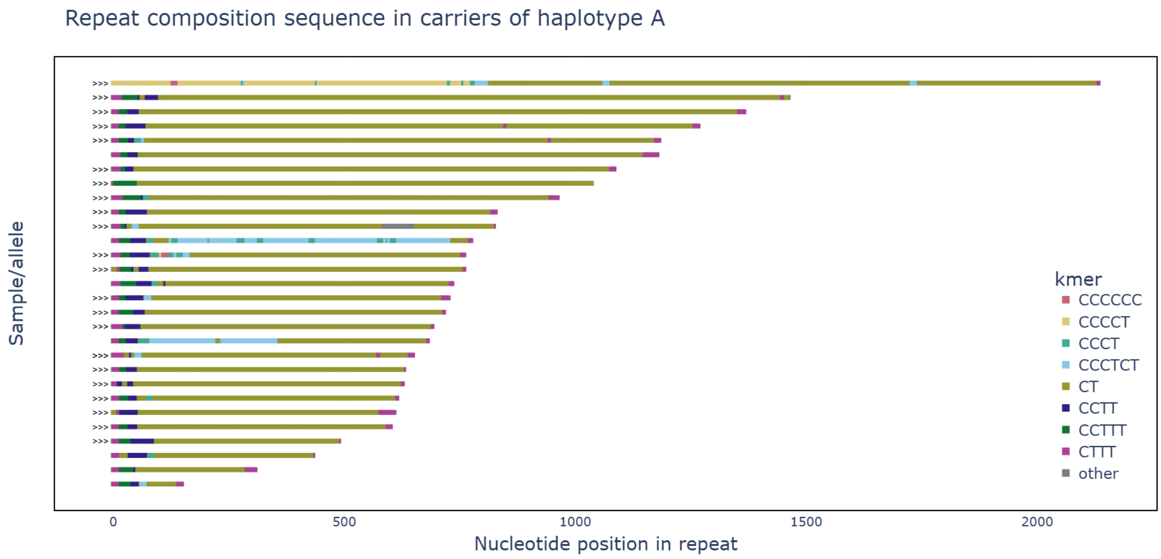

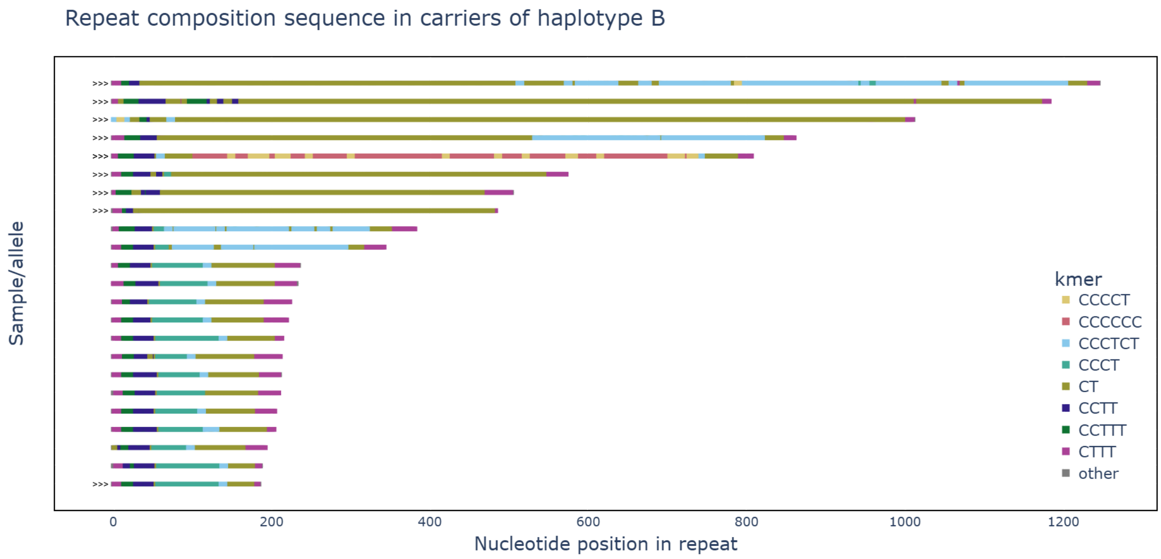

Supplementary Figure 11: These plots, generated with aSTRonaut show the repeat sequence for carriers of haplotype A (top) or haplotype B (bottom). Colors represent the observed motifs, and >>> annotations in front of the trace indicate an aFTLD-U patient. As the frequency of haplotype B in the general population is ~5%, the one aFTLD-U patient with haplotype B showing a short CT expansion reminiscent of those observed in non-aFTLD-U subjects presumably inherited the haplotype by chance and has a disease etiology different than chr15q14.

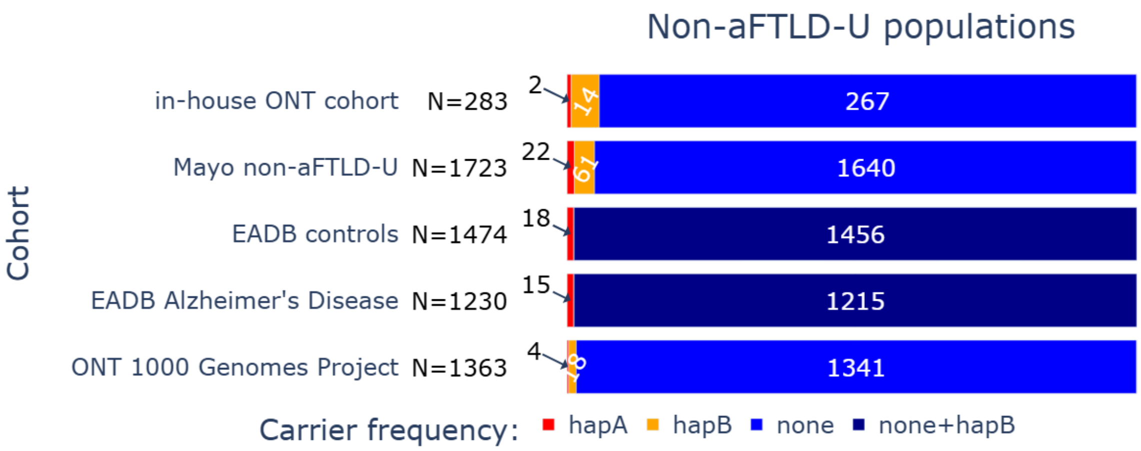

Supplementary Figure 12: Overview of risk haplotype carrier frequencies in the additional cohorts of non-aFTLD-U subjects included in the analyses. The Horizontal bar chart represents frequencies (as shown by color coding) and the absolute number of carriers with each haplotype, with individuals with missing genotypes removed. No haplotype B frequency was available for the EADB control individuals and Alzheimer's Disease patients, as rs148687709 could not be imputed reliably.

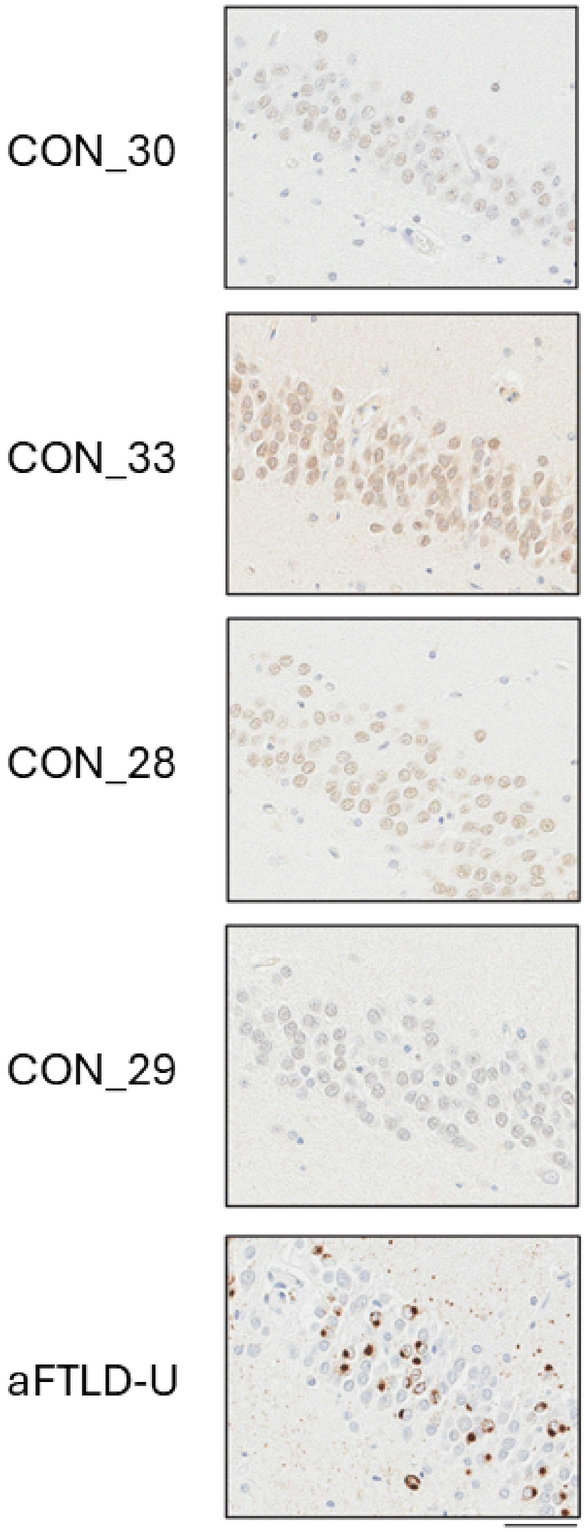

Supplementary Figure 13: TAF15 immunohistochemistry in non-aFTLD-U *GOLGA8A* repeat expansion carriers (TAF15 antibody 1:500, A300-308, Bethyl Laboratories). TAF15 inclusions characteristic of aFTLD-U were not detected in the dentate gyrus of four non-aFTLD-U subjects carrying chromosome 15q14 risk haplotype A. CON_28, CON_29, and CON_30 carried haplotypes with >190 CT-dimers, whereas CON_33 was classified as having a short CT repeat (sample labeling in reference to **Supplementary Table 5**). Scale bar, 10um.

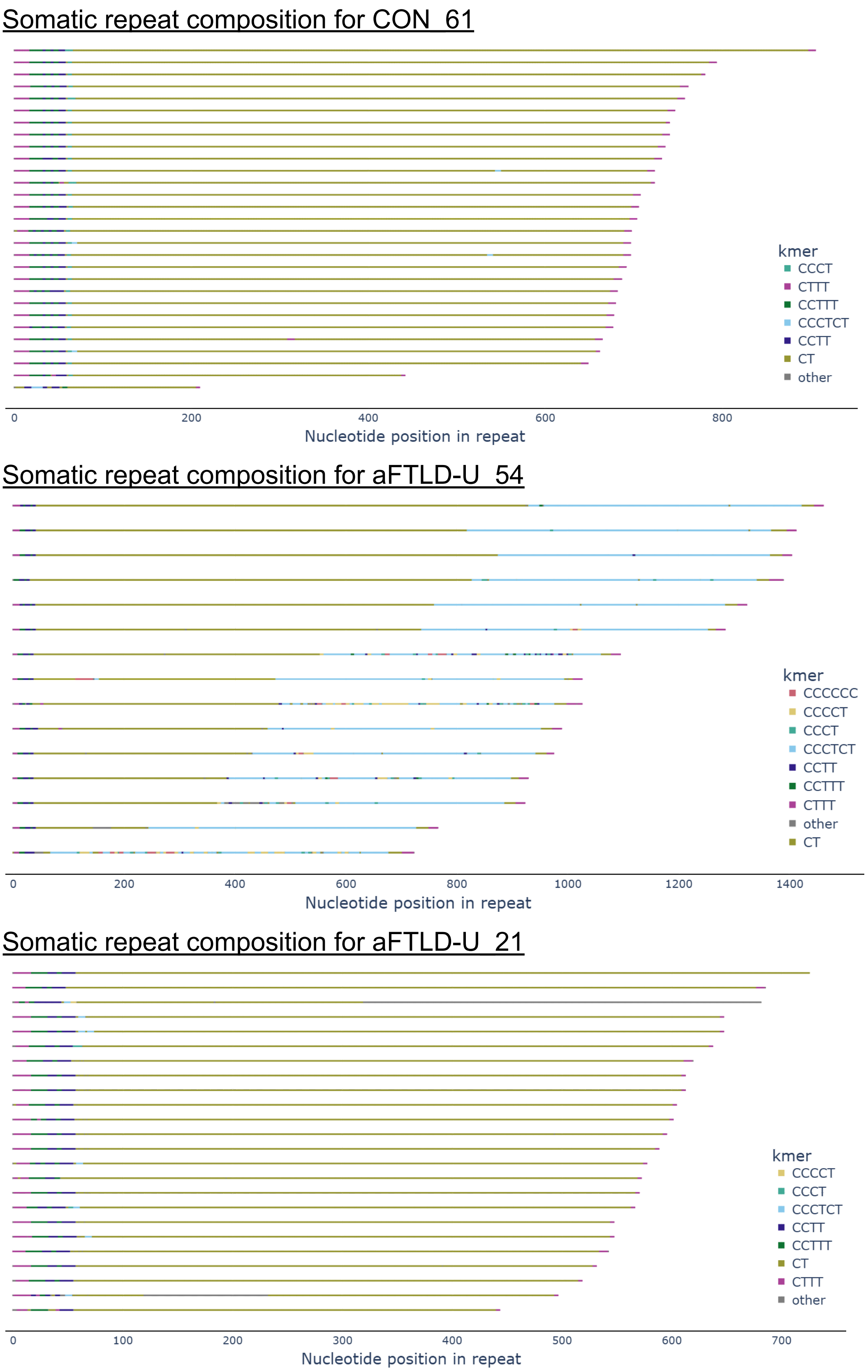

Supplementary Figure 14: Examples of aSTRonaut plots showing the somatic variation within one sample, with every horizontal line indicating a separate read (sample labeling in reference to **Supplementary Table 5**).

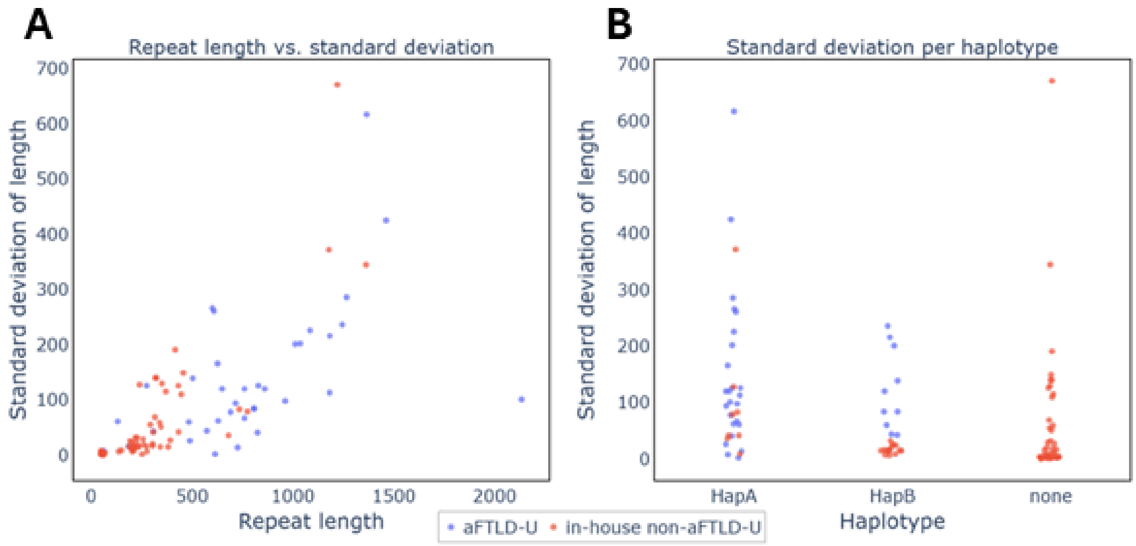

Supplementary Figure 15: These plots show the somatic variation on the repeat length for every expanded allele, regardless of repeat composition and including CCTT tetramer expansions **(A)** scatter plot of the standard deviation of the repeat length vs. repeat length **(B)** strip plot of the standard deviation of the repeat length per haplotype and disease status.

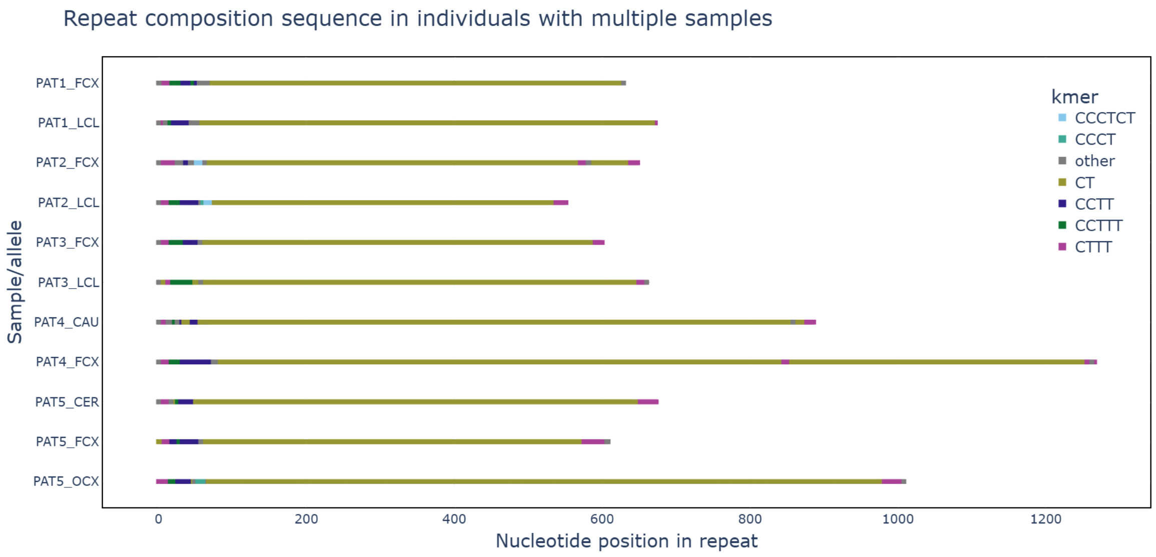

Supplementary Figure 16: aSTRonaut plot showing the repeat composition in samples for which DNA from multiple tissues was analyzed by long-read sequencing. Colors represent the observed motifs. FCX = frontal cortex, LCL = lymphoblastoid cell line, CAU = caudate, CER = cerebellum, and OCX = occipital cortex.

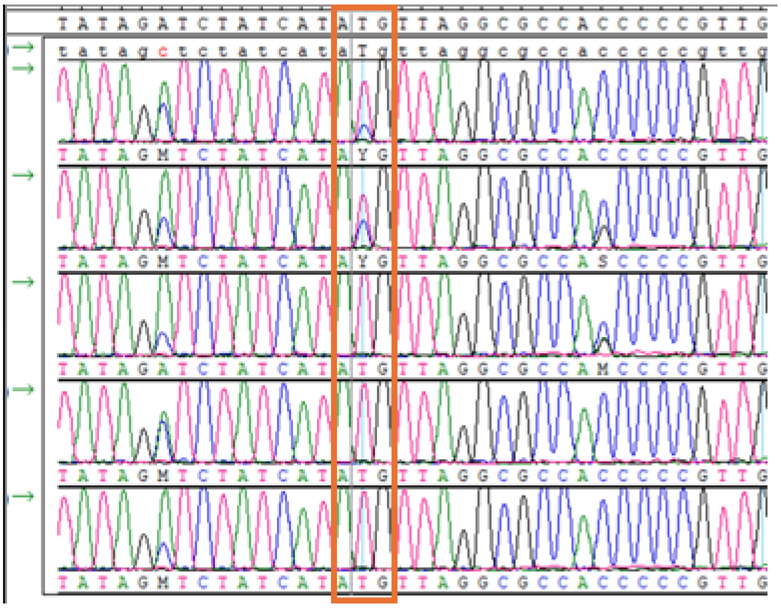

Supplementary Figure 17: Sanger sequencing of rs148687709, to be interpreted as a tetraploid position due to lack of primers specific for *GOLGA8A*, therefore also amplifying the paralogous sequence at *GOLGA8B*. The relevant position is highlighted with an orange box. This example shows, from top to bottom, a heterozygous carrier (1/4th of the peak is the alternative allele), a heterozygous carrier who also carries a deletion of the GOLGA8A-B locus (1/3th of the peak is the alternative allele) and three non-carriers.

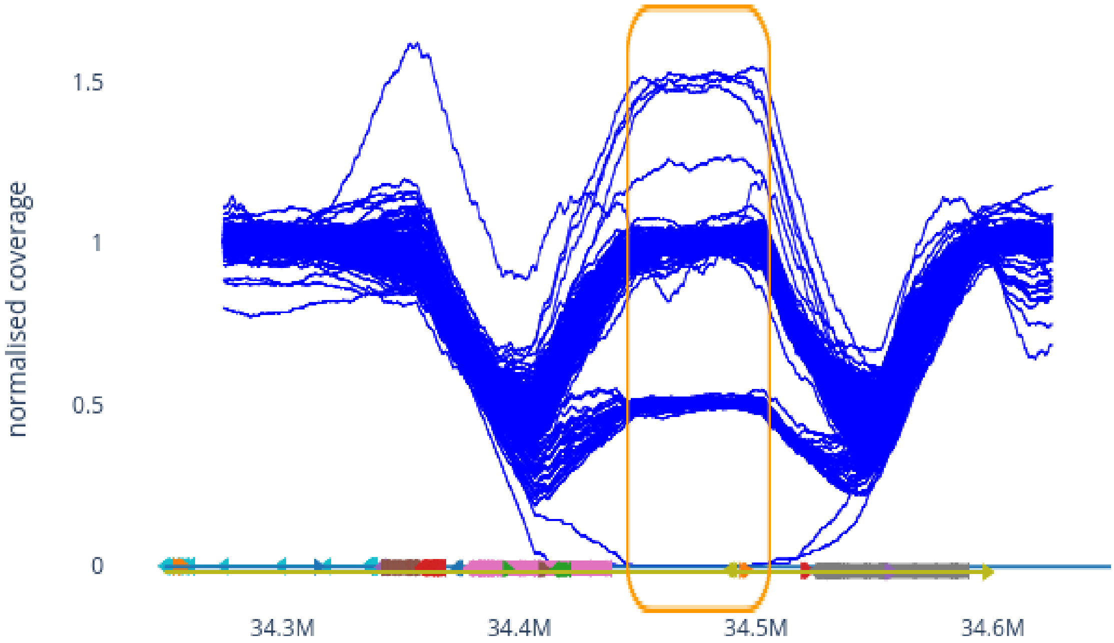

Supplementary Figure 18: Copy number analysis in *GOLGA8A-B* locus. Every blue line indicates an individual with data from short-read sequencing data. The bottom track indicates gene annotation, with the *GOLGA8A* gene in pink and *GOLGA8B* in grey. Coverage is determined using mosdepth, with normalization to a copy-neutral interval. The read alignment in the segmental duplications is typically ambiguous and can not be used for copy number estimation. A normalized coverage level in the fragment between *GOLGA8A* and *GOLGA8B* (orange box) of 1 indicates a diploid copy number, 0.5 is a heterozygous deletion carrier, etc.
