# Supplementary material for: A repeat expansion in GOLGA8A is a major risk factor for atypical frontotemporal lobar degeneration with ubiquitin-positive inclusions
Source: Nat Genet. 2026 Mar 12;58(4):726–36. doi: 10.1038/s41588-026-02537-7 (PMC13083237; doi:10.1038/s41588-026-02537-7)
Supplement: Supplementary file 2 — Reporting Summary [file 41588_2026_2537_MOESM2_ESM.pdf]

nature portfolio

Corresponding author(s): Rosa Rademakers

Last updated by author(s): Jan 19, 2026

Reporting Summary

Nature Portfolio wishes to improve the reproducibility of the work that we publish. This form provides structure for consistency and transparency in reporting. For further information on Nature Portfolio policies, see our Editorial Policies and the Editorial Policy Checklist.

Statistics

For all statistical analyses, confirm that the following items are present in the figure legend, table legend, main text, or Methods section.

n/a	Confirmed	
☐	☒	The exact sample size (*n*) for each experimental group/condition, given as a discrete number and unit of measurement
☐	☒	A statement on whether measurements were taken from distinct samples or whether the same sample was measured repeatedly
☐	☒	The statistical test(s) used AND whether they are one- or two-sided<br>*Only common tests should be described solely by name; describe more complex techniques in the Methods section.*
☐	☒	A description of all covariates tested
☐	☒	A description of any assumptions or corrections, such as tests of normality and adjustment for multiple comparisons
☐	☒	A full description of the statistical parameters including central tendency (e.g. means) or other basic estimates (e.g. regression coefficient) AND variation (e.g. standard deviation) or associated estimates of uncertainty (e.g. confidence intervals)
☐	☒	For null hypothesis testing, the test statistic (e.g. *F*, *t*, *r*) with confidence intervals, effect sizes, degrees of freedom and *P* value noted<br>*Give P values as exact values whenever suitable.*
☒	☐	For Bayesian analysis, information on the choice of priors and Markov chain Monte Carlo settings
☒	☐	For hierarchical and complex designs, identification of the appropriate level for tests and full reporting of outcomes
☐	☒	Estimates of effect sizes (e.g. Cohen's *d*, Pearson's *r*), indicating how they were calculated

Our web collection on statistics for biologists contains articles on many of the points above.

Software and code

Policy information about availability of computer code

Data collection

Short read sequencing data was processed using the Mayo Genome GPS v4.0 pipeline, and mapped to the human reference sequence (GRCh38 build) using the Burrows-Wheeler Aligner, and local realignment around indels was performed using the Genome Analysis Toolkit (GATK). Variant calling was performed using GATK HaplotypeCaller followed by variant recalibration (VQSR) according to the GATK best practices.

The sequencing data was base called with guppy (v6.7.3) or dorado (v7.1.4, v7.2.13, v7.3.11 and v7.4.13). The data was processed using snakemake workflows (github.com/wdecoster/chr15q14). Reads were aligned to the GRCh38 reference genome (GCA_000001405.15_GRCh38_no_alt_analysis_set) with minimap2 (v2.24), followed by sorting reads by coordinate and conversion to CRAM format with samtools (v1.16.1). The data quality was assessed with cramino (v0.14.5). Reads were phased with longshot (v0.4.5). SVs were called using Sniffles2 (v2.5.3) and SNVs with Clair3 (v1.0.2). The coverage of the unique sequence between GOLGA8A and GOLGA8B in the human reference genome was quantified using mosdepth (v0.3.8).

Ultra-long nanopore sequencing was basecalled using dorado (v7.3.11), filtered for reads longer than 25kb using chopper (v0.8.0) and assembled with hifiasm (v0.24.0-r703), followed by SV calling with svim-asm (v1.0.3).

Tandem repeats of interest were genotyped with STRdust (v0.11.7), the length of all human tandem repeats was determined using inquiSTR (v0.13.0).

Data analysis

Variant filtering for short-read sequencing data prior to GWAS was done using bcftools and R, and gene annotation of variants was performed using ANNOVAR (version2016Feb01). Relatedness was calculated with KING. The SNV-level analyses were performed using REGENIE and

PLINK (v.00a23LM2).

Python scripts, and jupyter notebooks for the long-read data analysis are available at https://github.com/wdecoster/chr15q14. VCF files were parsed with cyvcf2 (v0.30.16), visualization was done using Plotly (v5.14.1) and aSTRonaut (v1.0). Statistical analysis of the copy number was performed for carriers of the deletion allele using a Fisher exact test as implemented in scipy (v1.15.1). We developed STR_regression.R (v1.6) (github.com/wdecoster/inquiSTR/scripts/STR_regression.R) for running association testing of tandem repeat lengths. Precision and recall was calculated using scikit-learn (v1.6.1) with confidence intervals calculated using bootstrapping as implemented in scipy (v1.15.1).
Fragment lengths ffrom capillary electrophoresis were visualized using the in-house developed traci software (v1.1.0) (github.com/derijkp/traci).

For manuscripts utilizing custom algorithms or software that are central to the research but not yet described in published literature, software must be made available to editors and reviewers. We strongly encourage code deposition in a community repository (e.g. GitHub). See the Nature Portfolio guidelines for submitting code & software for further information.

Data

Policy information about availability of data

All manuscripts must include a data availability statement. This statement should provide the following information, where applicable:

- Accession codes, unique identifiers, or web links for publicly available datasets
- A description of any restrictions on data availability
- For clinical datasets or third party data, please ensure that the statement adheres to our policy

Individual-level data regarding participants' phenotype and sex, their GOLGA8A repeat characteristics (length, composition, CT dimer count, etc.), and the locus copy number are available in Supplementary Table 3. A dynamic version of the 'aSTRonaut' plot 3D is available at https://wdecoster.github.io/chr15q14/anonymized_aSTRonaut_all.html. Summary data on all tested variants of the GWAS analysis is available at https://my.locuszoom.org/gwas/943037/ and in GWAS catalog database under accession code GCST90726626. Short-read whole genome sequencing data from 23 aFTLD-U and 19 controls from Phase I were previously deposited in the dbGAP platform as part of the dataset with accession code phs003309 [https://www.ncbi.nlm.nih.gov/projects/gap/cgi-bin/study.cgi?study_id=phs003309.v1.p1]. For the 23 aFTLD-U patients, access is restricted: 9 can be for General Research Use, 1 is for Health/Medical/Biomedical research only, and 13 are for 'Disease-Specific (Neurodegenerative Disorders)' research only. The dbGAP ids of the patients included in this study can be found in Supplementary Table 6. The 19 controls can also be used for Disease-Specific (Neurodegenerative Disorders) research only. Access can be obtained by applying for dbGaP Authorized Access via https://view.ncbi.nlm.nih.gov/dbgap-controlled. The remaining 1285 controls from Phase I are from Mayo Clinic and are not available due to data sharing constraints related to the participants' consent form. The genetic data for the 38 aFTLD-U patients from Phase II are also not part of dbGAP accession phs003309 and not available due to data sharing constraints related to the participants' consent form. The gVCF genetic data from ADSP used in Phase II is available through restricted to not-for-profit organizations, access can be obtained by applying at https://dss.niagads.org/. The long-read sequencing data from HG01514 is available at ENA under the accession id ERR15094524.

Research involving human participants, their data, or biological material

Policy information about studies with human participants or human data. See also policy information about sex, gender (identity/presentation), and sexual orientation and race, ethnicity and racism.

Reporting on sex and gender	Sex is used as a covariate in all appropriate analyses, i.e. the GWAS and the number of males and females included in each population is summarized in Supplementary Table 4. Self-reported sex was used in all instances, and confirmed by genetic analysis where possible.
Reporting on race, ethnicity, or other socially relevant groupings	For the collection of FTLD-FET patients as part of this study no a priori selection was performed on race. All patients were self-reported Caucasian except for one aFTLD-U patient of Asian ancestry (who was excluded when calculating group frequencies and reported separately), and this was confirmed by genetic analysis. Overall, individuals included in the GWAS (including control individuals) with <70% European ancestry based on Admixture analysis were removed.
Population characteristics	Multiple populations have been used in this study and their population characteristics have been summarized in Supplementary Table 4.
Recruitment	We aimed to include all known FTLD-FET patients. For this we established an international consortium through inquiries at brain banks focused on neurodegenerative disease research and by contacting authors of relevant publications. All patients or their next of kin provided consent to participate in research studies in accordance with the Declaration of Helsinki and local ethics review board standards. We do not anticipate the study design resulted in relevant biases.
Ethics oversight	The ethics committee of the University Hospital Antwerp and the University Antwerp approved the study, collection of biomaterials was approved by local ethical committees of the participating sites.

Note that full information on the approval of the study protocol must also be provided in the manuscript.

Field-specific reporting

Please select the one below that is the best fit for your research. If you are not sure, read the appropriate sections before making your selection.

☒ Life sciences ☐ Behavioural & social sciences ☐ Ecological, evolutionary & environmental sciences

For a reference copy of the document with all sections, see nature.com/documents/nr-reporting-summary-flat.pdf

Life sciences study design

All studies must disclose on these points even when the disclosure is negative.

Sample size	No sample size calculation was performed, instead the maximal available number of patients at the time of the study was included.
Data exclusions	In the GWAS standard quality control metrics on the variant and individual level were applied. All analyses of the tandem repeat alleles required a minimal length of 100bp for inclusion of the individual, for all other analyses no data was excluded
Replication	We aimed to reproduce the key findings described in our study, wherever possible. First, after identifying a significant locus on chr15q14 using a small cohort of patients, we expanded the patient cohort through additional sample recruitment and performed a second GWAS, which confirmed the initial results. Next, we tested cohorts of additional (non-overlapping) patients and controls to replicate the observed frequencies of the disease associated haplotypes. Due to the small sample size statistics was not performed in these additional cohorts but frequencies were compared and found to be consistent. We further replicated the very low frequency of disease-associated haplotypes in several additional cohorts of control individuals and patients with other neurodegenerative diseases. As an alternative approach, we also performed a GWAS of aFTLD-U with the length of short tandem repeats as continuous predictor variables in our long-read sequencing cohort, which identified the same risk locus/STR further strengthening the disease locus. For the estimation of the optimal cut-off in terms of repeat length and composition to differentiate aFTLD-U patients from controls, we proposed two different approaches and report the precision and recall of both. All available data was used to provide the most accurate predictions and thus we could not replicate these cut-offs as part of this study. Future studies, in which additional patient and control cohorts are sequenced, should be used to replicate or refine our cut-offs.
Randomization	Due to the limited number of patients all individuals were included and compared against control individuals. Age and sex were included in the analysis as covariates to control for non-matched cohort characteristics.
Blinding	All short and long-read sequencing experiments and data processing were performed blinded, including the determination of the repeat size and the composition. Results were unblinded for interpretation of the findings.

Reporting for specific materials, systems and methods

We require information from authors about some types of materials, experimental systems and methods used in many studies. Here, indicate whether each material, system or method listed is relevant to your study. If you are not sure if a list item applies to your research, read the appropriate section before selecting a response.

Materials & experimental systems

n/a	Involved in the study	
☐	☒	Antibodies
☐	☒	Eukaryotic cell lines
☒	☐	Palaeontology and archaeology
☒	☐	Animals and other organisms
☒	☐	Clinical data
☒	☐	Dual use research of concern
☒	☐	Plants

Methods

n/a	Involved in the study	
☒	☐	ChIP-seq
☒	☐	Flow cytometry
☒	☐	MRI-based neuroimaging

Antibodies

Antibodies used	FUS (used 1:500 dilution, 11570-1-AP, Proteintech Group) or (used 1:200 dilution; HPA008784; Sigma, St. Louis. MO) or (used 1:500 dilution, A300-302A, Bethyl Laboratories) or (used 1:200, aa1-50, Novus) and TAF15 (used 1:500 dilution, A300-308, Bethyl Laboratories),
Validation	The FUS antibodies are routinely used in immunohistochemistry (IHC) to diagnose human patients with FET pathology. The FUS antibody from Proteintech ismost widely used and has reactivity to human FUS and was validated by the manufacturer to work on IHC. 35 publications have previously used this antibody in IHC applications. The TAF15 antibody is a validated antibody (according to the manufacturer's website) which means that the antibody passed multiple pillars of antibody validation. It has reactivity to human TAF15 and has been used in IHC applications in previous publications. In our study these antibodies were only used for a qualitative assessment of FUS and TAF pathology to confirm the diagnosis of aFTLD-U.

Eukaryotic cell lines

Policy information about cell lines and Sex and Gender in Research

Cell line source(s)	in-house generated EBV-transformed lymphoblastoid cell lines were included for two individuals (one male, one female, siblings) and one EBV-transformed lymphoblastoid cell line was requested from Coriell (one female).
Authentication	Full genome analysis was performed to confirm the identity of the cell line and compared with other available samples of the individuals and relatives.

Mycoplasma contamination	Lines were not tested for mycoplasma contamination
Commonly misidentified lines (See ICLAC register)	None

Plants

Seed stocks	N/A
Novel plant genotypes	N/A
Authentication	N/A
